# Ultralow dark current in near-infrared perovskite photodiodes by reducing charge injection and interfacial charge generation

**DOI:** 10.1038/s41467-021-27565-1

**Published:** 2021-12-14

**Authors:** Riccardo Ollearo, Junke Wang, Matthew J. Dyson, Christ H. L. Weijtens, Marco Fattori, Bas T. van Gorkom, Albert J. J. M. van Breemen, Stefan C. J. Meskers, René A. J. Janssen, Gerwin H. Gelinck

**Affiliations:** 1 Molecular Materials and Nanosystems, Institute for Complex Molecular Systems, Eindhoven University of Technology, P.O. Box 513, 5600 MB Eindhoven, The Netherlands; 2 Integrated Circuits, Departments of Electrical Engineering, Eindhoven University of Technology, P.O. Box 513, 5600 MB Eindhoven, The Netherlands; 3 TNO at Holst Centre, High Tech Campus 31, 5656 AE Eindhoven, The Netherlands; 4 Dutch Institute for Fundamental Energy Research, De Zaale 20, 5612 AJ Eindhoven, The Netherlands

**Keywords:** Electronic devices, Photonic devices

## Abstract

Metal halide perovskite photodiodes (PPDs) offer high responsivity and broad spectral sensitivity, making them attractive for low-cost visible and near-infrared sensing. A significant challenge in achieving high detectivity in PPDs is lowering the dark current density (*J*_D_) and noise current (*i*_n_). This is commonly accomplished using charge-blocking layers to reduce charge injection. By analyzing the temperature dependence of *J*_D_ for lead-tin based PPDs with different bandgaps and electron-blocking layers (EBL), we demonstrate that while EBLs eliminate electron injection, they facilitate undesired thermal charge generation at the EBL-perovskite interface. The interfacial energy offset between the EBL and the perovskite determines the magnitude and activation energy of *J*_D_. By increasing this offset we realized a PPD with ultralow *J*_D_ and *i*_n_ of 5 × 10^−8^ mA cm^−2^ and 2 × 10^−14^ A Hz^−1/2^, respectively, and wavelength sensitivity up to 1050 nm, establishing a new design principle to maximize detectivity in perovskite photodiodes.

## Abstract (web summary)

In lead‐tin halide perovskite photodiodes, the interfacial energy offset at the charge transport layer controls the dark current. In this work, by increasing this offset, the authors demonstrates a photodiode with ultralow dark and noise currents and high near‐infrared sensitivity.

## Introduction

Minimizing the dark current density (*J*_D_) of emerging thin film flexible photodiodes is essential for near-infrared (NIR) sensing and imaging^1–3^. Metal halide perovskites are solution processable semiconducting materials that have attracted extensive interest for their remarkable photovoltaic properties^4,5^, but are likewise promising candidates for photodiodes. Their high carrier mobility, long electron-hole diffusion lengths, and low exciton binding energy^6–8^ enable high and fast responsivity to light^9–13^. Additional benefits include low processing temperature, and an optical absorption spectral range that can be tuned through structural and compositional modification^14–16^. Notably, alloying lead halide perovskites with tin extends the detection range further into the NIR, with absorption wavelengths up to 1050 nm^17^.

However, to date Pb and mixed Pb–Sn-based perovskite photodiodes (PPDs) have suffered from relatively high dark currents. This unwanted property has been attributed to the susceptibility of divalent Sn to oxidation^18^, charge injection from the contacts as well as structural and compositional imperfections in the material, leading to pinholes, trap states, and grain boundary leakage^19^. Collectively, these factors increase *J*_D_ and the device noise current level (*i*_n_) thus limiting the specific detectivity (D^*^), a key figure of merit that describes the smallest detectable signal.

Over recent years, significant efforts have been devoted to minimizing the dark current in thin film perovskite photodiodes. Besides material driven strategies including the use of antioxidant additives^20,21^, prevention of shunt paths^22^, control of film crystallization^23^, and passivation of traps^24,25^, the use of hole-blocking and electron-blocking layers (HBLs and EBLs) has been demonstrated to be critical for the control of *J*_D_^26–29^. These charge-blocking layers increase the energy barrier for undesired charge injection, which occurs from the electrodes into the light-absorbing semiconductor layer under reverse bias. Previous work on PPDs with such device architecture and similar operating principle have reported dark current densities as low as 10^−6^ to 10^−7^ mA cm^−2^, typically measured at −0.5 V^30–33^. In addition, the HBL and EBL are designed to enable extraction of photogenerated charges from the active layer and therefore generally have good electron and hole transporting properties.

Despite the success of these charge-blocking layers, a comprehensive understanding of their role in suppressing the dark current in perovskites has yet to be fully developed. While the measured *J*_D_ decreases with increasing energetic barrier heights, the drop does not follow the thermionic emission model, as will be shown below. Instead, the experimentally observed dark current density usually reaches a lower limiting value that is typically orders of magnitude higher than the expected intrinsic bulk thermal-generated dark current density (*J*_0_). Identifying the causes of such discrepancy would provide crucial insights for effective dark current suppression and thus increasing detectivity.

Here we study *J*_D_ in Pb and mixed Pb–Sn based halide PPDs with different organic EBLs. By varying the perovskite composition, i.e., the Pb to Sn ratio, and the EBL, we find that the dark current originates from a thermal charge generation process at the EBL-perovskite interface. We show that under −0.5 V reverse bias *J*_D_ depends on the energetic barrier *Φ* defined as the energy difference between the conduction band minimum (CBM) of the perovskite and the highest occupied molecular orbital (HOMO) of the EBL. By analyzing the temperature dependence of *J*_D_, we find that this energy difference *Φ* corresponds to the thermal activation energy (*E*_a_) of *J*_D_. For all PPDs analyzed in this work, the measured *J*_D_ scales exponentially with *Φ* and values lower than 10^−7^ mA cm^−2^ are achieved at room temperature for *Φ* > 1 eV. The noise current (*i*_n_) is also found to scale with *J*_D_, and thus with *Φ*. Hence, the role of the EBL in controlling the noise level of PPDs is related to its HOMO energy, and lowering that energy to increase *Φ* will reduce *i*_n_ and *J*_D_. We demonstrate the validity of this design rule by fabricating a NIR perovskite photodiode that features a record-low dark current density of 5 × 10^−8^ mA cm^−2^ and affords a sub-microsecond response time and a state-of-the-art specific detectivity of 2.5 × 10^12^ Jones. Our findings will fuel the development of novel, even more refined device architectures that further decrease dark current and increase specific detectivity, as well as integration of these exciting materials in a variety of different electronic and medical applications.

## Results

### Dark current in visible and NIR perovskite photodiodes

Perovskite photodiodes that absorb light in the visible and in the NIR were fabricated using different Pb:Sn perovskite compositions. The device is built on a glass substrate with a patterned indium tin oxide (ITO) electrode that is covered with the EBL, for which we first use poly[bis(4-phenyl)(2,4,6-trimethylphenyl)amine] (PTAA). Mid- and narrow-bandgap FA_0.66_MA_0.34_Pb_(1–*x*)_Sn_*x*_I_3_ perovskites (FA is formamidinium, MA is methylammonium) are used as the light-absorbing semiconducting layer. The diodes are completed with a double layer of C_60_ and bathocuproine (BCP) as HBL and an Ag electrode. The device structure (Supplementary Fig. 1a) has a SiN layer that covers the perimeter of the ITO electrode to minimize leakage currents^34^. A detailed description of the fabrication is provided in the Methods section. When illuminated, these photodiodes exhibit high EQEs (measured at a bias of −0.5 V, with peaks above 75% and approaching 65% at 940 nm, for narrow-bandgap compositions) and a linear response to light intensity (Supplementary Fig. 2).

**Fig. 1:**
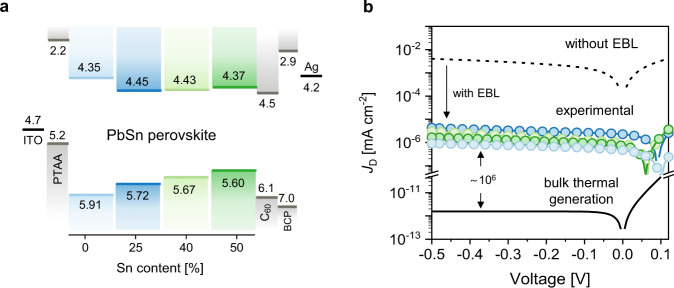
Energy diagram and dark current of perovskite photodiodes. **a** Schematic energy band diagram for mixed FA_0.66_MA_0.34_Pb_(1–*x*)_Sn_*x*_I_3_ perovskite photodiodes. Uncertainties in the energy levels are depicted in Supplementary Fig. 3. **b** Experimental (circles colored with light blue (*x* = 0), dark blue (*x* = 0.25), light green (*x* = 0.40) and dark green (*x* = 0.50)) and simulated (black lines) *J − V* characteristics in dark. Simulations were performed in absence (dashed line) and presence (solid line) of the PTAA electron blocking layer. Simulation parameters are presented in Supplementary Table 1 and Supplementary Note 1.

The relevant energy level diagram of these photodiodes for FA_0.66_MA_0.34_Pb_(1–*x*)_Sn_*x*_I_3_ perovskites with *x* = 0, 0.25, 0.40, and 0.50 is shown in Fig. 1a. The diagram shows the bandgap (*E*_g_) determined from UV-vis-NIR spectroscopy (Supplementary Fig. 1b), the energy of the valence band maximum (VBM) (*E*_V_) determined from UV photoelectron spectroscopy (UPS) (Supplementary Fig. 3), and the energy of the CBM (*E*_C_) calculated from *E*_C_ = *E*_V_ + *E*_g_. Figure 1a shows that energy level alignment of the PTAA and C_60_/BCP layers block direct injection of electrons from the ITO and holes from the Ag contacts under reverse bias, but facilitate extraction of photogenerated charges. To explore the influence of energy level alignment, Fig. 1b shows the current density−voltage (*J − V*) characteristics in the dark of the four PPDs, with different bandgaps, with an emphasis on the reverse bias region relevant to PPDs. We limited this bias to −0.5 V, because at more negative voltages perovskites suffer from ion migration which are known to cause degradation of the device^35^. When measured in a *J − V* sweep, the *J*_D_ at −0.5 V is in the range of 10^−5^ to 10^−6^ mA cm^−2^. When no EBL is used, a much higher value of ~10^−2^ mA cm^−2^ is found (Supplementary Fig. 4), because the injection barrier *Φ*_inj_, for a PPD without EBL (i.e., the energy difference between the work function of ITO (4.7 eV) and the CBM of FA_0.66_MA_0.34_Pb_0.5_Sn_0.5_I_3_) is only 0.35 eV. This effectively illustrates the role of the EBL in reducing *J*_D_.

Steady-state values of *J*_D_ under a constant applied −0.5 V bias (Supplementary Fig. 5) are 4 × 10^−7^ mA cm^−2^ (*x* = 0), ∼2.5 × 10^−6^ mA cm^−2^ (*x* = 0.25 and *x* = 0.40), and 8 × 10^−7^ mA cm^−2^ (*x* = 0.50) and lower than from *J − V* sweeps, because capacitive contributions are absent. Notably, these values are among the lowest dark current densities reported for visible and NIR perovskite-based photodiodes^23,36–38^. Furthermore, *J*_D_ appears to be unrelated to the oxidation of Sn^2+^ to Sn^4+^, because no consistent increase of *J*_D_ is observed when increasing the Sn percentage^18^.

To understand these experimental dark current densities, it is of interest to compare them to the expected intrinsic values. Assuming thermionic emission, the injection current (*J*_inj_) at reverse bias would scale exponentially with barrier *Φ*_inj,_ as

1 $${J}_{{{{{{\rm{inj}}}}}}}={A}{{T}}^{2}\exp \left[-\frac{q{\varPhi }_{{{{{{\rm{inj}}}}}}}}{{k}_{{{{{{\rm{B}}}}}}}T}\right]$$

where *Φ*_inj_ is the difference between the work function of ITO and the energy of lowest unoccupied molecular orbital (LUMO) of PTAA for electron injection, and between the work function of Ag and the HOMO energy of BCP for hole injection, respectively. *k*_B_ is the Boltzmann constant, *q* is the elementary charge, *T* is the temperature, and *A* is the pre-exponential factor known as Richardson constant^39^. Using drift-diffusion simulations that include thermionic emission we modelled the dark current density. These simulations reveal that for sufficiently large *Φ*_inj_ (from ITO to EBL LUMO and from Ag to HBL HOMO), injection currents are negligible (solid line in Fig. 1b) and *J*_D_ ≈ *J*_0_, where *J*_0_ arises from the bulk thermal excitation across the bandgap of the material. According to the drift-diffusion simulations, the intrinsic *J*_0_ is *ca*. 1 × 10^−12^ mA cm^−2^ (for the lowest *E*_g_ where *x* = 0.5, i.e., Pb_0.5_Sn_0.5_). Consequently, compositions with *x* < 0.5 and thus with wider bandgaps would exhibit even lower intrinsic dark current density, because ${J}_{0}\propto \exp (-{E}_{{{{{{\rm{g}}}}}}}/{k}_{{{{{{\rm{B}}}}}}}T)$. Furthermore, the simulations confirm that without an EBL *J*_D_ would be much higher (≈5 × 10^−3^ mA cm^−2^, dashed line in Fig. 1b). It is important to note that the experimentally observed *J*_D_ is six orders of magnitude higher than the intrinsic current (*J*_0_) expected in an ideal diode in which charge injection is inhibited (Fig. 1b). The observed discrepancy suggests the existence of an alternative dark current generation mechanism in real devices. At reverse voltages, such mechanism prevails over the bulk thermal generation and hinders the suppression of experimental *J*_D_ with blocking layers. Understanding this discrepancy is a fundamental step in lowering the dark current density of PPDs and thus maximizing their detectivity.

### Thermal activation energy of the dark current

**Fig. 2:**
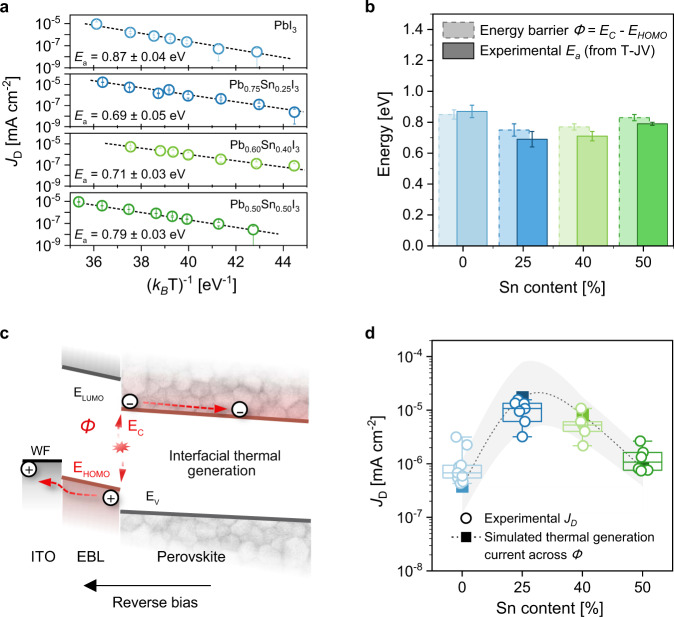
Analysis of the origin of dark current. **a** Temperature dependence of *J*_D_ at *V* = − 0.5 V for the four perovskite photodiodes. Open circles are experimental data, whiskers represent standard deviation. Linear fits according to *J*_D_ ∝ exp(−*E*_a_/*k*_B_*T*) are shown by dashed lines, standard errors are also reported. **b** Comparison between experimental activation energies *E*_a_ (bars with solid outline, whiskers are standard error) and energetic barriers (*E*_C −_ *E*_HOMO_, bars with dashed outline, whiskers indicate the uncertainty of energy levels) for the different Pb:Sn compositions. **c** Schematic representation of the thermal charge generation mechanism at the EBL-perovskite interface. **d** Comparison between the statistical distribution of the experimental *J*_D_ (open circles, with boxplots showing median (center line), 25th and 75th percentiles (box limits) and 5th and 95th percentiles (whiskers)) and the simulated dark current density (see Supplementary Fig. 9) resulting from interfacial generation for different values of *Φ* (squares) with uncertainty (gray area) at −0.5 V for Sn contents *x* = 0, 0.25, 0.40, and 0.50. Simulation details are presented in Supplementary Note 1.

To explore the origin of the experimental dark current density, we determined how *J*_D_ at a constantly applied −0.5 V bias varies with temperature (see “Methods” section and Supplementary Fig. 6). Figure 2a shows clear Arrhenius-type behavior for all compositions investigated, leading to thermal activation energies of *E*_a_ of 0.87 eV (*x* = 0), 0.69 eV (*x* = 0.25), 0.71 eV (*x* = 0.40), and 0.79 eV (*x* = 0.50) for the dominant source of dark current. Notably, these experimental activation energies are substantially lower than the energetic barriers for injection of electrons (*Φ*_inj,e_ = 2.5 eV) or holes (*Φ*_inj,h_ = 2.8 eV) between the metal contacts and the charge-blocking layers under reverse bias. Furthermore, for each PPD the activation energy *E*_a_ is smaller than *E*_g_ by 0.4 to 0.7 eV, thus highlighting that *J*_D_ does not scale with *E*_g_. This implies that well-established mechanisms such as charge injection and bulk thermal generation cannot be the dominant contribution to the experimental dark current densities, which must instead originate from a less energetically demanding process.

One possible origin of experimental *J*_D_ may be the effect of trap states that lie within the bandgap. Trap states can influence the device dark current, modify its temperature dependence, and thus affect *E*_a_. This is because the sub-bandgap states facilitate the bulk thermal charge generation, which requires electrons in the valence band or occupied intragap states (i.e., deep or shallow traps) to be thermally excited into the conduction band. Although the influence of trap states on *J*_D_ has been widely observed in inorganic and more recently in OPDs^3,40–42^, we demonstrate that trap states are unlikely to account for the observed *J*_D_ in these PPDs. We calculated the dark current density in the radiative limit (*J*_0_^rad^) and its activation energy (*E*_a_^EQE^) from the spectral overlap integral between the black body spectrum at different temperatures and the experimental (sub-bandgap) EQE spectrum (Supplementary Note 2). For the Pb:Sn perovskites, small EQE signals (≤10^−7^) were measured below the Urbach tail at the perovskite band-edge that can be attributed to defect states (see Supplementary Fig. 7a). Interference affects the sub-bandgap EQE as seen when comparing the sub-bandgap EQEs of opaque and semi-transparent PPDs (see Supplementary Fig. 7b). Calculating *J*_0_^rad^ from the EQE provides an estimated *E*_a_^EQE^ of 1.23 eV when integration is done over Urbach tail only, and an *E*_a_^EQE^ of ~0.65 eV when the entire experimental EQE spectrum is considered (Supplementary Fig. 8). The values are virtually identical for opaque and semi-transparent PPDs. An activation energy of 0.65 eV is close to the energetic position of mid-bandgap trap states (*E*_g_/2). While mid-bandgap trap states minimize the energy required for thermal excitation^43^, there is no clear correlation between *E*_g_/2 and the *E*_a_ measured from the temperature dependence of *J*_D_. The latter is significantly higher. Given that the experimental EQE is close to the noise floor of the setup (~10^−9^) and that the spectral overlap with the black body spectrum is largest at photon energies lower than covered by the EQE spectrometer (>0.63 eV), we extended the calculation to a variety of sub-bandgap EQE spectral shapes that correspond to various hypothetical trap state distributions. In all cases the calculated dark current density has a substantially different temperature dependence, exhibiting *E*_a_^EQE^ even lower than 0.65 eV (Supplementary Fig. 8).

Instead, the measured values of *E*_a_ resemble very closely the energetic barriers *Φ*, defined as the difference between the conduction band energy of the perovskites (*E*_C_) and the HOMO energy of the EBL. At the interface with PTAA which has *E*_HOMO_ = −5.2 eV, *Φ* equals 0.85 eV (*x* = 0), 0.75 eV (*x* = 0.25), 0.77 eV (*x* = 0.40), and 0.83 eV (*x* = 0.50). Figure 2b compares *E*_a_ with the energy offset *E*_C_ − *E*_HOMO_ for each composition. Significantly, the histogram reveals close correspondence between the two energies, with differences of less than 0.1 eV. Energetically, this similarity correlates the EBL-perovskite interface and both the energy states (i.e., the CBM of the perovskite and the HOMO of the EBL) to *J*_D_. Interpreting the nature of this correlation is therefore required to understand how the energetic states involved determine dark current in PPDs.

### Thermal generation of dark current at the perovskite and organic EBL interface

Due to the energy level offset (Fig. 1a), charge generation at both organic-perovskite heterojunction interfaces requires less energy than the bandgap of either semiconductor. For the PPDs in this study, the smallest energetic barrier is at the EBL-perovskite interface (Fig. 1a) and originates from the relatively high *E*_HOMO_ of organic EBLs^44^. At a non-zero temperature, an electron in the HOMO level of the EBL can be thermally excited to the nearest available energy state, specifically the perovskite CBM. We propose this interfacial thermal charge generation mechanism as the origin of the experimental dark current. We suggest a model in which charge carriers are first generated at the EBL-perovskite interface via thermal energy and then collected at the respective contacts by the applied electric field thus generating a current. This mechanism is schematically depicted in Fig. 2c. Due to the energy offset at the heterojunction, the electron-hole pair easily dissociates into free charges^45^. Following dissociation, charge collection occurs efficiently by hole transport via the EBL and electron transport via the perovskite. The resulting dark current density is thus determined by interfacial thermal generation, and hence varies with the temperature and the amplitude of the interfacial barrier through the typical Boltzmann exponential relation ${J}_{{{{{{\rm{D}}}}}}}\propto \exp (-\varPhi /{k}_{{{{{{\rm{B}}}}}}}T)$, where *Φ* = *E*_C_ − *E*_HOMO_.

Following the method employed by Wang et al.^46^, in which blocking layers are treated as contacts with the assumption of no transport loss beyond their interface with the perovskite, we simulated the dark current density resulting from the interfacial charge generation for the four different perovskite photodiodes (Supplementary Fig. 9). All simulation parameters were kept constant except *E*_C_ (and thus *Φ*) and *E*_V_ (see Supplementary Note 1 and Supplementary Table 2 for details). The simulated current density corresponds well to the absolute magnitude and trends that were measured experimentally as function of Sn content (Fig. 2d). These results illustrate that the proposed mechanism is likely to be the dominant source of dark current in real devices, thus underlining the importance of energetic gap between the EBL HOMO and the perovskite CBM for *J*_D_. Notably, through this interfacial charge generation path, photons with energy < *E*_g_ might be optically absorbed and contribute to the sub-bandgap EQE signal. However, we cannot unambiguously assign the weak signals seen in the sub-bandgap EQE spectrum (Supplementary Fig. 7) to this direct optical interfacial transition.

### The influence of EBL HOMO on the dark current

**Fig. 3:**
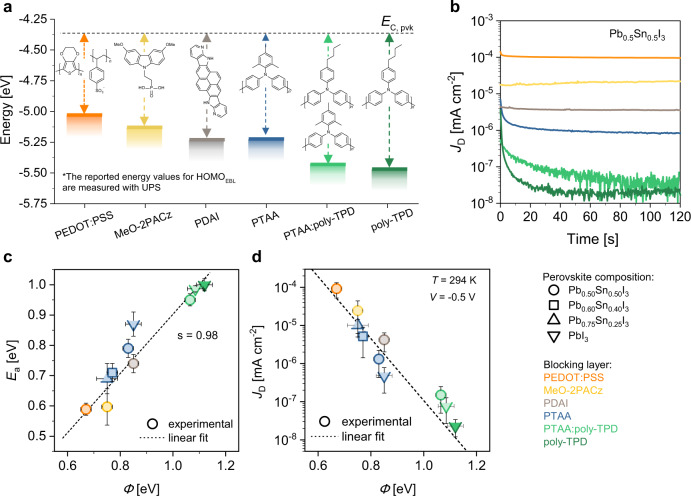
Effect of E_HOMO_ of the EBL on dark current. **a** HOMO levels of all electron blocking layers (*E*_C, pvk_ represents the CBM of FA_0.66_MA_0.34_Pb_0.5_Sn_0.5_I_3_ perovskite). **b** Reverse dark current densities of PPDs with different EBLs (and FA_0.66_MA_0.34_Pb_0.5_Sn_0.5_I_3_ perovskite). *J*_D_ is measured over time by applying a constant bias of −0.5 V. **c** Activation energy of *J*_D_, *E*_a_, plotted vs. the energetic difference *Φ* = *E*_C_ − *E*_HOMO_ for different EBL-perovskite systems, as indicated by the legend. Vertical whiskers indicates the standard error in fitting *E*_a_. **d** Experimental dark current density (measured at *V* = −0.5 V, room temperature) vs. *Φ* for the same combination of electron blocking layers and Pb:Sn compositions. In the interval plot, colored circles and whiskers are mean values and 95th percentile confidence interval, respectively. The horizontal whiskers in (**c**) and (**d**) represent the uncertainty of energy levels.

To further strengthen our hypothesis that the dark current is due to thermal charge generation at the EBL-perovskite interface, we study *J*_D_ for a range of different EBLs. Figure 3a depicts the HOMO energies and chemical structures of the EBL materials investigated: poly(3,4-ethylenedioxythiophene):polystyrene sulfonate (PEDOT:PSS), [2-(3,6-dimethoxy-9H-carbazol-9-yl)ethyl]phosphonic acid (MeO-2PACz), pyrenodi-(7-azaindole) (PDAI), PTAA, poly(4-butyl-*N,N*-diphenylaniline) (poly-TPD), and a PTAA:poly-TPD (1:1) mix^47–51^. All the chosen EBLs have LUMO energies above −3 eV. The HOMO levels, as determined by UPS (Supplementary Fig. 10), were selected to lie within the energy interval defined by the ITO work function (4.7 eV) and the perovskite VBM (−5.6 eV for Pb_0.5_Sn_0.5_) (Fig. 3a). An *E*_HOMO_ outside this energy range would introduce undesired charge extraction barriers and thus negatively affect the electrical response of the device to light.

Figure 3b shows *J*_D_, measured at −0.5 V, for each EBL/Pb_0.5_Sn_0.5_ combination (see Supplementary Fig. 12 for Pb-based devices). The *J − V* characteristics of these devices are provided in Supplementary Fig. 11. Lowering the HOMO energy increases *Φ* and decreases the experimental *J*_D_ by approximately 4 orders of magnitude for a HOMO energy difference of 0.5 eV. The extremes of this range are illustrated by devices made with PEDOT:PSS and poly-TPD, which show a *J*_D_ of 10^−4^ and ∼10^−8^ mA cm^−2^, respectively. Notably, the deep HOMO level of PTAA:poly-TPD and poly-TPD (~−5.45 eV) resulted in a *J*_D_ < 10^−7^ mA cm^−2^, demonstrating the efficacy of increasing *Φ* to reduce *J*_D_. To the best of our knowledge, no perovskite photodiodes have been reported with similarly low dark current density^30,32,33,36,38^. Figure 3b shows that for small *J*_D_’s, the *J*_D_ is reduces in the first tens of seconds. This effect is stronger at lower temperatures (see Supplementary Fig. 6) and, therefore, unlikely related to ion migration. We associate it with capacitive effects or built-up of space charge.

The activation energies *E*_a_, derived from analysis of temperature dependent *J*_D_ (Supplementary Fig. 13), have an approximately linear relationship with *Φ* (Fig. 3c). Since *E*_a_ ≈ *Φ*, we conclude that dark current is a function of the EBL HOMO. Furthermore, in Fig. 3d we illustrate the relationship between the (room temperature) dark current density and interfacial energy barrier. For the blocking layers and perovskite compositions investigated, the relation between *J*_D_ and *Φ* resembles an Arrhenius relationship typical of a thermally-activated process. This dependence is entirely compatible with the proposed description of the generation mechanism at the EBL-perovskite interface, highlighting the influence of the EBL energy levels.

### The influence of EBL HOMO on the noise current

**Fig. 4:**
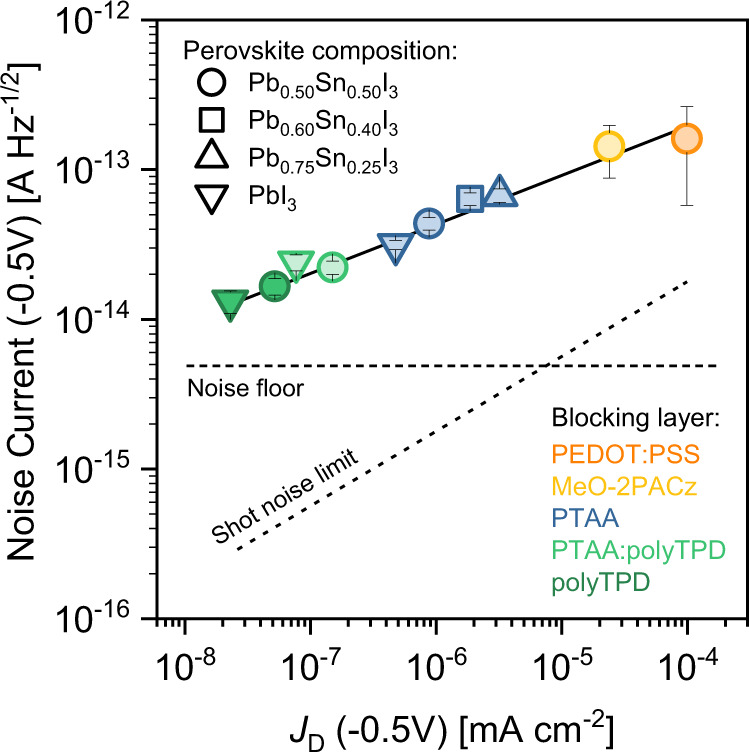
Correlation on the dark current density and the noise current. Noise current measured under reverse bias (−0.5 V) vs. dark current density of PPDs with different EBL-perovskite combinations, as indicated in the legend. Colored circles and whiskers are median values and standard deviation, respectively, extrapolated in the frequency region between 10–100 Hz (i.e., where noise is dominated by white noise) (see Supplementary Fig. 14). The shot noise limit is also plotted for comparison (dashed line).

The specific detectivity of a photodiode depends on the noise current^52^. It is thus imperative to investigate the relationship between dark current density and noise current of the EBL-perovskite systems studied above. The current noise spectral density, measured at reverse bias in the frequency interval *f* = 1 to 100 Hz, is presented in Supplementary Fig. 14. At low frequencies 1/*f* behavior is observed for PPDs exhibiting higher dark current density. Above 10 Hz, the frequency response tends to converge to a plateau value. The relation between the noise current *i*_n_ (averaged between 10 and 100 Hz) and *J*_D_, is shown in Fig. 4. As the residual dark current density decreases, the noise current also reduces, showing a frequency independent spectrum. For those combinations of EBL-perovskite with a large interfacial barrier *Φ*, such as poly-TPD or mixed PTAA:poly-TPD with *x* = 0 and *x* = 0.50 perovskite, noise current values of 1 − 2 × 10^−14^ A Hz^−1/2^ were achieved. Given the relation between *i*_n_ and *J*_D_, these measurements highlight the direct correlation between the noise current and *E*_HOMO_, and thus to the specific detectivity. Note that the total experimental noise current is on average one order of magnitude higher than the shot noise (*i*_n,s_) calculated from the dark current, according to the expression:

2 $${i}_{{{{{{\rm{n}}}}}},{{{{{\rm{s}}}}}}}=\sqrt{2qB{I}_{{{{{{\rm{D}}}}}}}}$$

where *I*_D_ is the dark current, *q* is the elementary charge, and *B* is the bandwidth. This suggests that other sources of white noise, such as thermal noise, also provide a significant contribution to the overall noise of the device.

### Formulating design rules for ultralow dark current NIR perovskite photodiodes

Since incorporating an EBL reduces direct charge injection but enhances thermal charge generation at the EBL-perovskite interface, its HOMO energy must be considered when designing PPDs. Perovskite interfaces are known to be sensitive to structural imperfections, dangling bonds, defects and trap states^53–55^, often making control of interfacial thermal charge generation difficult. As such, accurate tuning of blocking layer energy levels at the perovskite interface represents a relatively simple but very effective solution to control the dark current density.

**Fig. 5:**
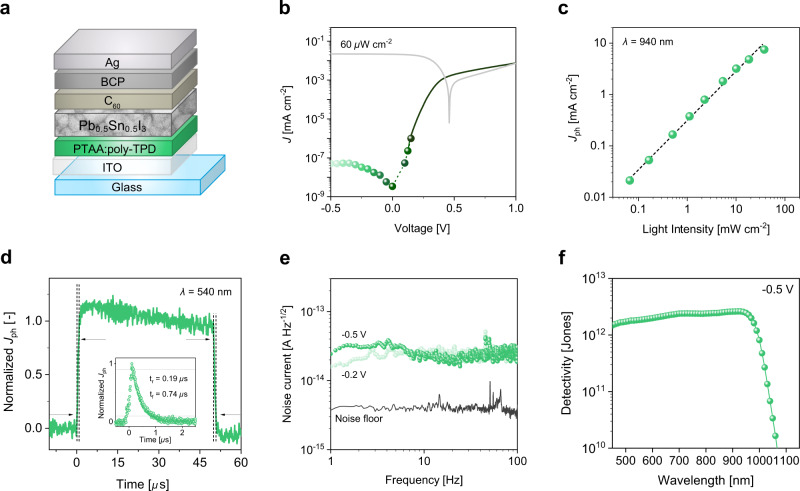
Photodiode performance of a Pb_0.5_Sn_0.5_I_3_ perovskite and with a PTAA:poly-TPD EBL. **a** Schematic device structure. **b** *J − V* characteristic in dark and under NIR light (940 nm); the current density is limited at high forward bias, i.e., outside the typical operating region of the photodiode, as result of the energetic hole injection barrier; solid circles are current density values derived from constant voltage measurements over time at discrete biases (Supplementary Fig. 15a). **c** Linearity plot measured at −0.5 V showing *J*_ph_ at different photon fluxes under NIR radiation (940 nm). **d** Normalized transient photocurrent response under low light intensity (540 nm, 0.8 mW cm^−2^) upon square light pulses (50 μs duration) and single peak pulses (6 ns, inset), exhibiting a rise (and fall) time, i.e., time for response vary from 10% (90%) to 90% (10%), lower than 1 μs. **e** Noise current vs. frequency at −0.2 and −0.5 V. **f** Detectivity of the photodiode at different wavelengths under reverse bias (−0.5 V).

Following the outlined design rule of maximizing *Φ*, we fabricated a NIR photodiode employing PTAA:poly-TPD as EBL and FA_0.66_MA_0.34_Pb_0.5_Sn_0.5_I_3_ as NIR-absorbing perovskite. A PTAA:poly-TPD blend was chosen over the pure poly-TPD layer to ensure an improved surface wettability for the perovskite layer while keeping unaltered the energetic position of the HOMO level, which is *ca*. −5.45 eV. The device architecture and key performance characteristics in dark and in NIR light (*λ* = 940 nm, 60 μW cm^−2^) are shown in Fig. 5. As expected, due to the maximized interfacial energetic barrier between the electron-blocking layer and the perovskite, the reverse dark current density of 5 × 10^−8^ mA cm^−2^ at −0.5 V is very low and in fact close to the detection limit of our instrument. The photocurrent *J*_ph_ produced by the diode is 28.5 mA cm^−2^ at 1 sun (integrated from the EQE spectrum at *V* = −0.5 V, see Supplementary Fig. 15b), corresponding to a *J*_ph_/*J*_D_ ratio of ∼10^9^ at −0.5 V. At larger reverse bias, *J*_ph_ shows minimal variation, indicating a virtually bias-independent extraction efficiency of photogenerated charges (Supplementary Fig. 15b). The *J*_ph_/*J*_D_ ratio decreases, however, more than four orders of magnitude for *V* < −1 V due to the increase of *J*_D_ (Supplementary Fig. 15c, d). At −0.5 V, both *J*_D_ and *J*_ph_ do not change significantly with time, pointing towards its good operational stability (Supplementary Fig. 15e). We note that these measurements were performed on a PPD that was stored for more than 12 months, indicating a promising long shelf life.

The EQE of the device in the NIR region is between 68% (at 850 nm) and 63% (at 940 nm), and then decreases to 20% at 1000 nm (Supplementary Fig. 16a). This corresponds to a peak of spectral responsivity (SR) at 940 nm of ∼0.5 A W^−1^, which is 2× higher than those reported for similar perovskite composition^21^. Notably, such high SR was achieved also using different EBLs (Supplementary Fig. 16b). The photo-response of the PPD to different light intensities is close to linear (*J*_photo_ ~ *I*^*α*^ with *α* = 0.95), producing photocurrent values ranging from 2.1 × 10^−2^ to 7.4 × 10^0^ mA cm^−2^ for light intensities ranging from ≈60 to 4 × 10^4^ µW cm^−2^, as shown in Fig. 5c. Dynamically, the photocurrent rapidly increases and decays in response to light pulses (540 nm, 50 μs duration), exhibiting a sub-microsecond rise/fall time of 0.79/0.88 μs (Fig. 5d), which reduces to 0.19/0.74 μs when illuminated by a single peak pulse (540 nm, 6 ns duration, inset Fig. 5d). The EBL material has no significant effect on the response speed of the photodiodes (Supplementary Fig. 16c): values range between 0.8–2.4 μs and are primarily limited by the large geometric capacitance. Microsecond-fast response is comparable to that of best visible PPDs and faster than NIR PPDs (Supplementary Table 3). The noise spectral density of the device measured at −0.2 and −0.5 V are independent of frequency (no sign of 1/*f* dependence in the low frequency region), with constant value of 2 × 10^−14^ A Hz^−1/2^ (Fig. 5e). Therefore, the diode’s noise is dominated by white noise. Moreover, since 1/*f* noise is generally associated to a (slow) carrier trapping and de-trapping process^56^, the frequency independent spectrum underlines the negligible influence of deep trap states. Finally, combining the measured noise current (*i*_n_) and spectral responsivity (SR), the specific detectivity D^*^ of the diode at −0.5 V was calculated to be 2 × 10^12^ Jones over the whole spectral range, with a maximum of 2.5 × 10^12^ Jones in the NIR region (at 940 nm), as represented in Fig. 5e. Detectivities of this order are comparable to those of commercial Si (4 × 10^12^ Jones)^57^ and GaP (2.3 × 10^12^ Jones)^58^ PDs, and among the highest reported for PPDs^13,21,30–33^, especially for the NIR. Under the often-used assumption of predominating shot noise, the photodiode would achieve a maximum D^*^ of 7 × 10^13^ Jones at 940 nm (Supplementary Fig. 16d). Despite being widely used in the literature, we consider this method to determine D^*^ less appropriate, because the total noise usually differs from the shot noise, as pointed out in Fig. 4. Overall, the PPD shows state-of-the-art performance, with an extremely low dark current density, noise current, a fast and linear photo-response, and an excellent responsivity and specific detectivity in the visible and NIR, also compared to recent related studies (see Supplementary Table 3).

## Discussion

For several narrow- and mid-bandgap PPDs, the experimental *J*_D_ exceeds by many orders of magnitude the intrinsic theoretical value *J*_0_, thus excluding thermal charge generation in the bulk of the perovskite as the main cause of *J*_D_ in absence of injection currents. We instead assert that thermal charge generation occurring at the interface between the EBL and the perovskite is the origin of the dark current density in PPDs. The energy barrier at this interface determines the achievable lower limit of *J*_D_. The activation energy *E*_a_ of *J*_D_ determined from its temperature dependence correlates with the interfacial energy barrier *Φ* = *E*_C_ − *E*_HOMO_ between the conduction band of the perovskite and the HOMO of the EBL. This suggests that other dark current producing mechanisms such as charge injection or bulk and trap-assisted generation are negligible in comparison. These conclusions are supported by drift-diffusion simulations that reproduce the magnitude and the trend of *J*_D_ when interfacial charge generation is included. Further evidence is given by characterization of multiple EBL-perovskite systems, which show that both *J*_D_ and its activation energy scale with *Φ*. Maximizing this barrier by using EBLs with a deeper HOMO enabled fabricating a PPD with an extremely low *J*_D_ of 5 × 10^−8^ mA cm^−2^ and a noise level of 2 × 10^−14^ A Hz^−1/2^, while keeping high responsivity to visible and NIR light along with sub-microsecond temporal response. We have thus revealed that while the EBL suppresses the injection current at the interface with the metal contact, it also actively participates to the generation of a detrimental dark current. This work thus provides new directions compared to existing optimization strategies for dark current minimization in PPDs, along with new design rules that account for the outlined interfacial charge generation process. The state-of-the-art performance will further accelerate the uptake of perovskite photodiode technology in a variety of different electronic and medical applications.

## Methods

### PPD fabrication

A 135 nm transparent ITO film was sputtered on glass and photolithographically structured to form the bottom electrode. Next, a 50 nm SiN layer was deposited and patterned via dry etch to cover the perimeter of the ITO bottom electrode. This SiN layer defines the active device area and helps to prevent leakage currents, as typically shown for a-Si diodes (Mulato et al.^34^), organic photodetectors^59,60^ and more recently also perovskite detectors^61^. Prior the deposition of the active layer stack, the substrates were cleaned with 30 min of UV-ozone treatment. After that, the devices were transferred to a N_2_ glovebox. The electron blocking layers were then processed as follows. A solution of PTAA (Solaris Chem) in toluene (2 and 20 mg mL^−1^) was spin coated at 5000 rpm for 35 s and then annealed at 100 °C for 10 min. Poly-TPD film was deposited from solution (in chlorobenzene, 20 mg mL^−1^) with the same spin coating and annealing conditions. For the PTAA:poly-TPD mix, PTAA (10 mg) and poly-TPD (10 mg) were dissolved in dichlorobenzene (1 mL), then spin coated at 2500 rpm for 40 s and annealed at 130 °C for 35 min. MeO-2PACz (TCI) solution in anhydrous ethanol (0.35 mg mL^−1^) was deposited by spin coating at 3000 rpm for 30 s and annealed at 100 °C for 10 min. The PEDOT:PSS film was deposited via spin coating from aqueous dispersion (PVP Al 4083, Heraeus Clevios) and subsequently patterned via dry etch (O_2_ plasma) to specifically cover the pixel active area. PDAI was thermally evaporated under high vacuum (~10^−7^ mbar)^62^. For the sequential solution-processed Pb–Sn hybrid perovskite precursor solutions, a 1.2 mmol PbI_2_ (Sigma-Aldrich, beads, 99.999% trace metal basis) and SnI_2_ (Sigma-Aldrich, beads, 99.99% trace metals basis) mixture was dissolved in *N,N-*dimethylformamide (DMF, 0.876 mL) (Sigma-Aldrich, anhydrous 99.8%) and dimethyl sulfoxide (DMSO, 0.0864 mL) (Sigma-Aldrich, anhydrous 99.9%). In the solution mixture, the molar fraction of SnI_2_ (*x*) was varied between 0, 0.25, 0.40, an 0.50, and 10 mol% SnF_2_ (Sigma-Aldrich, 99%) with respect to SnI_2_ was added^63^. All solutions were filtered beforehand with a PTFE 0.22 µm filter. Formamidinium iodide (53.48 mg) (FAI, Greatcell Solar) and methylammonium iodide (25.6 mg) (MAI, Greatcell Solar) mixture was dissolved in 1 mL of 2-propanol (Sigma-Aldrich, anhydrous 99.5%) at 60 °C. For the perovskite surface passivation, ammonium thiocyanate (NH_4_SCN) (Sigma-Aldrich) in 2-propanol (1 mg mL^−1^) was dynamically spin coated at 5000 rpm for 30 s. Finally, the device was completed by evaporating the electron transport layers, C_60_ (SES Research, 20 nm) and BCP (Lumtec, 8 nm), and the top metal electrode (Ag, 100 nm) under a high vacuum (~10^−7^ mbar).

### Ultraviolet photoelectron spectroscopy

The UPS measurements were performed in a VG EscaLab II system with a base pressure of 10^−8^ Pa using He–I radiation (21.22 eV) and a bias of −6 V^64^.

### PPD characterization

Room temperature *J − V* characteristics were measured between −0.5 V and +1.5 V with voltage steps of 5 mV, using an Agilent 4155C semiconductor parameter analyzer connected to manual probes in a N_2_-filled glovebox. To avoid capacitance and charging effects and obtain a more accurate value of reverse *J*_D_ at −0.5 V, current density was monitored under the applied constant bias voltage over time until a steady-state value was reached. Temperature dependent *J*_D_ measurements were performed in a cryostat under vacuum (*P* = 10^−4^ mbar) with a probe station connected to a Keithley (2636A) source meter and programmed with a custom written LabView code. Temperature was controlled using a LakeShore 336 temperature controller. The EQE was measured using a custom-made setup consisting of a tungsten-halogen lamp, a monochromator (Oriel, Cornerstone 130), a chopper, a pre-amplifier (Stanford Research Systems SR570) and a lock-in amplifier (Stanford Research Systems SR830 DSP). For the measurement, the devices were kept sealed in a N_2_-filled box equipped with a quartz window, on which a circular aperture (1 mm diameter) was applied. The calibration of the EQE signal was made through a reference silicon solar cell. The standard deviation of this setup is less than 0.005 electron/photon (in the range 350–1050 nm of wavelengths). Within the same setup, the light intensity-dependent photo response of the PPD was measured, driving a 940 nm LED between 1 and 1000 mA and using a reference Si photodiode (Thorlabs FDS100) for calibration. For the EQE in the sub-bandgap region, an Oriel 3502 light chopper, Cornerstone 260 monochromator (CS260-USB-3-MC-A), a Stanford Research SR 570 preamplifier, a Stanford Research SR830 lock-in amplifier, and a 250 W tungsten-halogen lamp were used. A series of long pass filters with increasing cut-on wavelengths was placed between the lamp and monochromator to remove stray light during the measurement. The photodiode was kept in a nitrogen atmosphere during the measurement. Calibrated Si and InGaAs photodiodes were used to determine incident light intensity. The transient photocurrent (TPC) was measured using a pre-amplifier (Stanford Research Systems SR570) and a digital oscilloscope (Tektronix TDS5052B). Light pulses were generated by a green LED (530 nm) connected to a wave-function generator (Agilent 33250A). The incident light was focused on the device through an optical focal lens (Thorlabs) and a circular aperture (active pixel area of 1 mm in diameter). Noise measurements were performed at room temperature and in dark conditions, exploiting a battery-powered current to voltage conversion readout circuit developed with off-the-shelf components. The setup is further arranged in a metal enclosure to shield the device under test from electromagnetic interference. The photodiode (with device area of 1 mm^2^) was connected by means of two probes and triaxial cables to a trans-impedance amplifier (TIA) implemented with the operation amplifier Analog Devices (ADA4530). An adjustable DC voltage source was applied to the non-inverting terminal of the TIA to modify the biasing of the device. The output of the TIA was fed to an active bandpass amplifier (Analog Devices AD8065, with in-band voltage gain of 100 V/V) and read out by a dynamic signal analyzer (HP35670A).

## Supplementary information

Supplementary Information

## Data Availability

All relevant data in this study are available from the corresponding authors upon request. Source data are provided with this paper.

## Source data

Source data
