# Supplementary material for: Ultralow dark current in near-infrared perovskite photodiodes by reducing charge injection and interfacial charge generation
Source: Nat Commun. 2021 Dec 14;12:7277. doi: 10.1038/s41467-021-27565-1 (PMC8671406; doi:10.1038/s41467-021-27565-1)
Supplement: Supplementary file 1 — Supplementary Information [file 41467_2021_27565_MOESM1_ESM.pdf]

Supplementary Information

Ultralow dark current in near-infrared perovskite photodiodes by reducing charge injection and interfacial charge generation

Riccardo Ollearo[1], Junke Wang[1], Matthew J. Dyson[1], Christ H. L. Weijtens[1], Marco Fattori[2], Bas T. van Gorkom[1], Albert J. J. M. van Breemen[3], Stefan C. J. Meskers[1], René A. J. Janssen[1,4], Gerwin H. Gelinck[1,3]

[1] Molecular Materials and Nanosystems, Institute for Complex Molecular Systems, Eindhoven University of Technology, P.O. Box 513, 5600 MB Eindhoven, The Netherlands.

[2] Integrated Circuits, Departments of Electrical Engineering, Eindhoven University of Technology, P.O. Box 513, 5600 MB Eindhoven, The Netherlands.

[3] TNO at Holst Centre, High Tech Campus 31, 5656 AE Eindhoven, The Netherlands.

[4] Dutch Institute for Fundamental Energy Research, De Zaale 20, 5612 AJ Eindhoven, The Netherlands.

Supplementary Figures

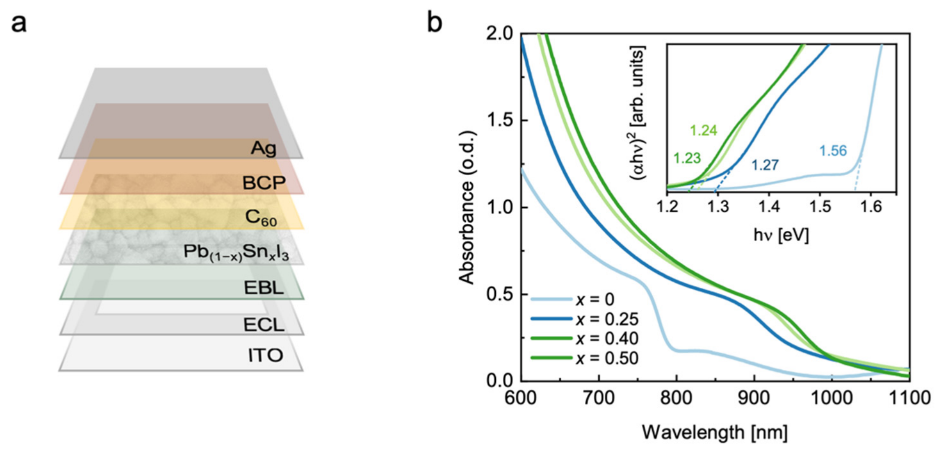

Supplementary Fig. 1. PPD device structure and active layer absorption spectra. a, Schematic device structure of PPD. **b**, Absorption spectrum of $FA_{0.66}MA_{0.34}Pb_{(1-x)}Sn_xI_3$ perovskite films with x = 0, 0.25, 0.40, 0.50. The apparent absorption in the interval 800-1000 nm is due to interference and scattering of light between the top of the film and the glass surface. The inset shows the Tauc plots for the same perovskite compositions. The optical bandgap was determined by extrapolating the straight-line portion of the plot.

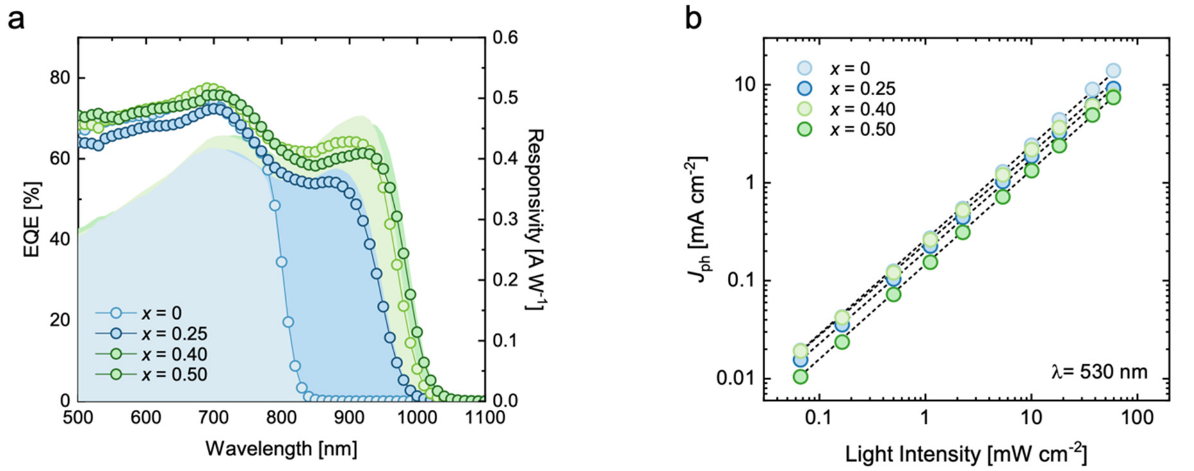

Supplementary Figure 2. Light response of photodiodes. a, External quantum efficiency (EQE) (colored circles) and spectral responsivity (SR) (colored area) vs. wavelength measured at –0.5 V for PTAA-$FA_{0.66}MA_{0.34}Pb_{(1-x)}Sn_xI_3$ PPDs. **b**, Linearity plot measured at −0.5 V showing J_{ph} vs. light intensity for the same photodiodes.

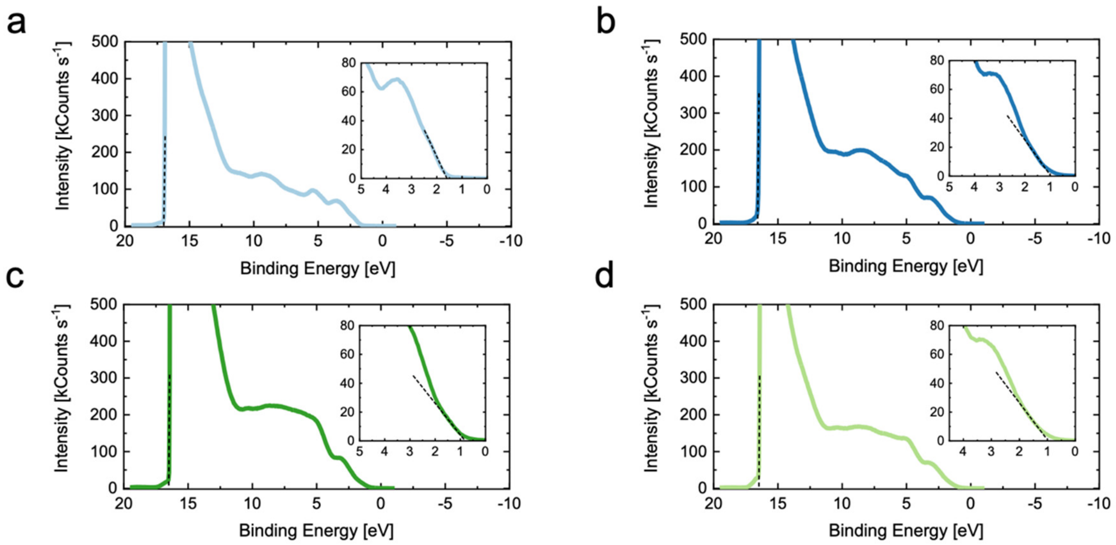

Supplementary Fig. 3. UPS measurements of $FA_{0.66}MA_{0.34}Pb_{(1-x)}Sn_xI_3$ perovskites films. a, x = 0. **b**, 0.25. **c**, 0.40, **d**, 0.50. The VBM energy is determined from $E_{VBM} = E_{He-I} - \Delta E$, where E_{He-I} = 21.22 eV is the excitation energy and ΔE is the energy difference between the secondary-electron emission onset and the onset of the UPS spectrum. The resulting VBMs with uncertainty are: 4.35 ± 0.03 eV (x = 0), 4.45 ± 0.04 eV (x = 0.25), 4.43 ± 0.02 eV (x = 0.40) and 4.37 ± 0.02 eV (x = 0.50).

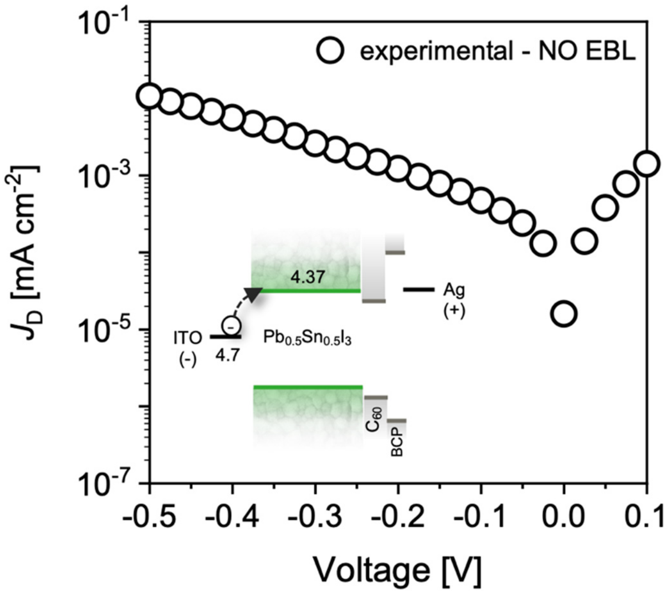

Supplementary Fig. 4. Current density-voltage (*J-V*) characteristic of a $FA_{0.66}MA_{0.34}Pb_{0.5}Sn_{0.5}I_3$ PPD without EBL. Inset shows the corresponding energy diagram with schematic representation of charge injection from ITO to active layer.

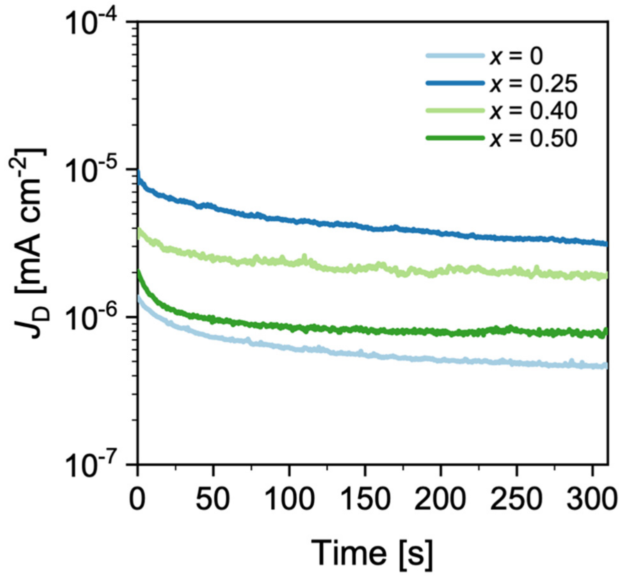

Supplementary Fig. 5. **Current density transient** for $FA_{0.66}MA_{0.34}Pb_{(1-x)}Sn_xI_3$ perovskites PPDs measured over time at an applied constant bias of –0.5 V at room temperature (with PTAA as EBL). Steady-state J_D was extracted from the close-to-constant value at 240-300 s.

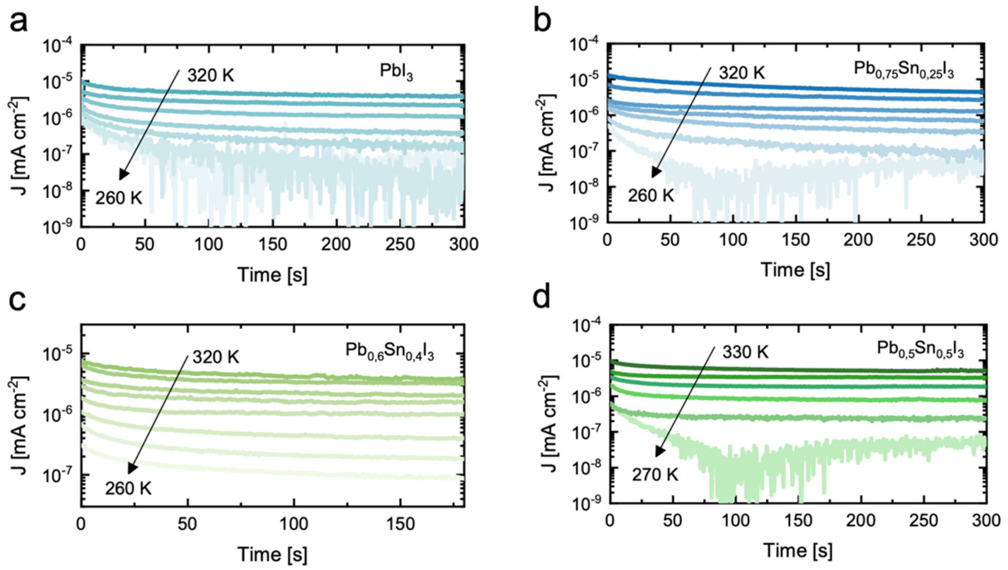

Supplementary Fig. 6. Time dependence of J_D as function of temperature for PTAA−$FA_{0.66}MA_{0.34}Pb_{(1-x)}Sn_xI_3$ PPDs. a, x = 0. **b**, x = 0.25. **c**, x = 0.40. **d**, x = 0.50. Dark current density was recorded at V = –0.5 V until a constant value was reached (and extrapolated as mean value with standard deviation from the last 60 s of measurement). The investigated temperature range was determined by the device stability and the instrument's detection limit (~ 10^{-9} mA cm^{-2}).

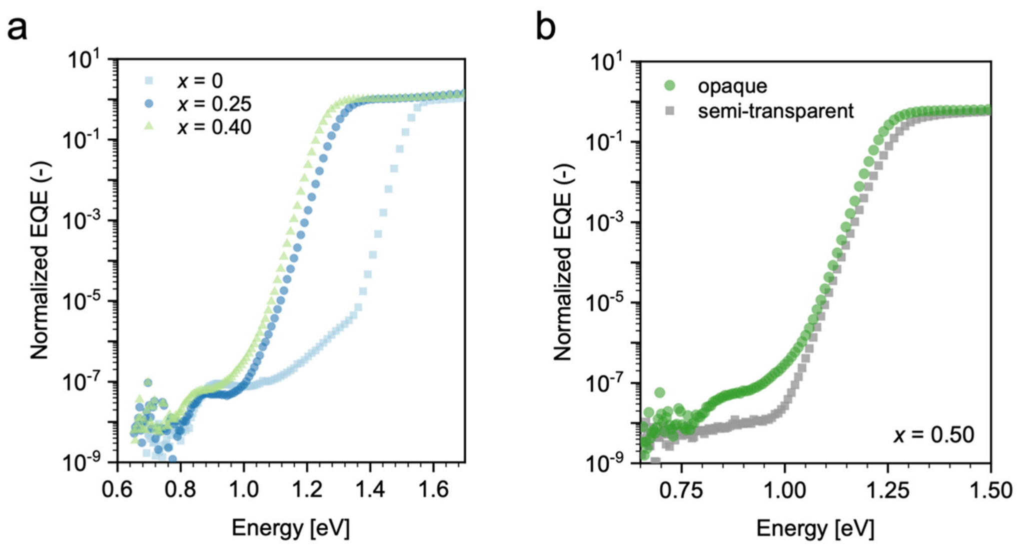

Supplementary Fig. 7. Sub-bandgap EQE spectra for $FA_{0.66}MA_{0.34}Pb_{(1-x)}Sn_xI_3$ PPDs. a, For $x = 0$, 0.25, and 0.40. **b**, for $x = 0.50$ (b). In panel b, the spectra obtained with two device configurations with opaque (top Ag) and semi-transparent (top ITO) electrodes are shown. This shows that the minor signal between 0.8 and 1.0 eV is enhanced by optical interference due to photons being reflected by the silver layer. This interference is less when an optically transparent ITO top contact is used. The intensity of sub-bandgap EQE is enhanced by this effect.

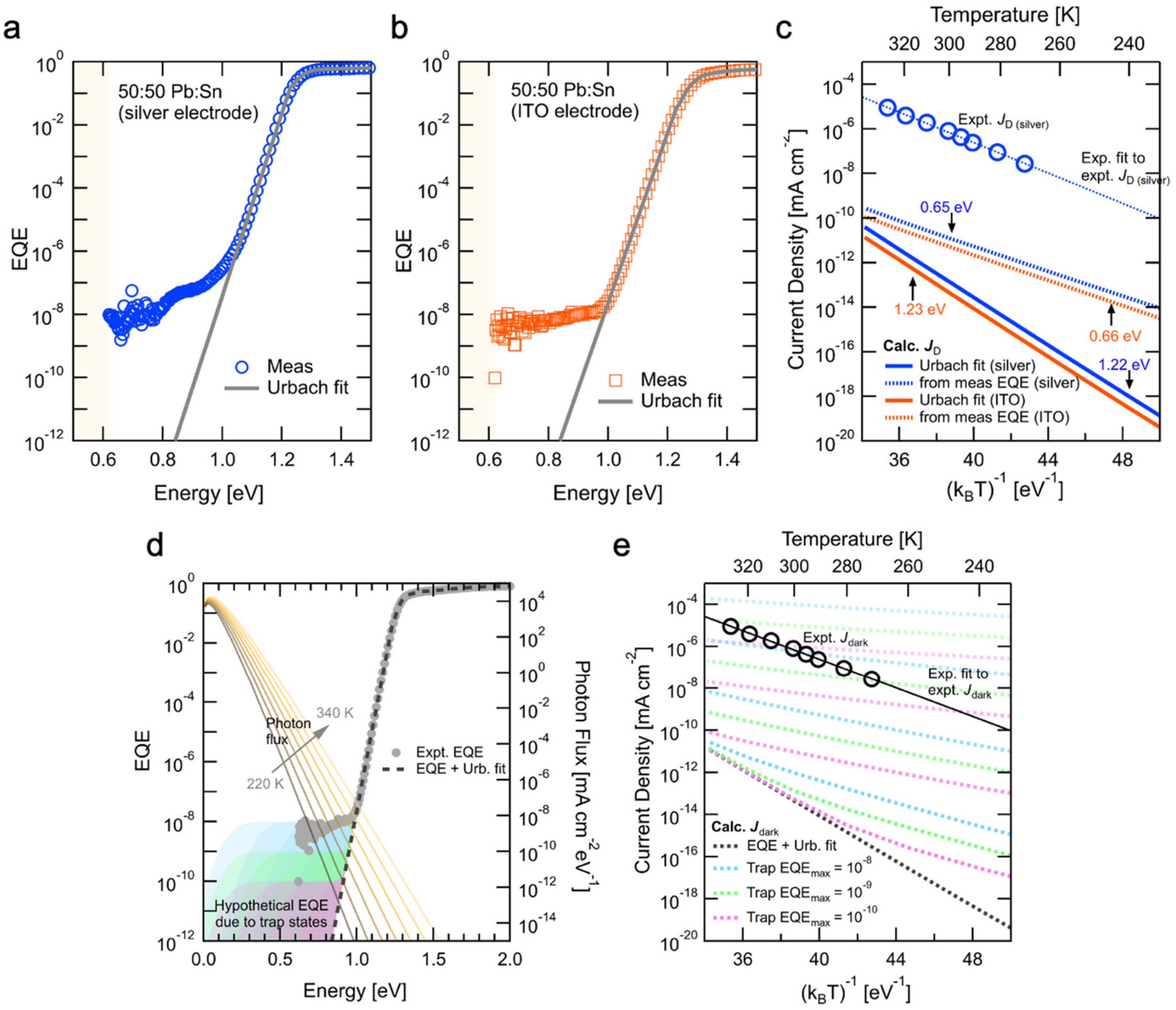

Supplementary Fig. 8. Calculated J_D from overlap integrals of thermal black-body photon flux with extrapolated EQE spectra. **a**, Sub-bandgap EQE spectrum of $FA_{0.66}MA_{0.34}Pb_{0.50}Sn_{0.50}I_3$ PPD with top Ag electrode (see Supplementary Figure 1a). The solid line is a fit of the Urbach tail. **b**, Sub-bandgap EQE spectrum of a semi-transparent $FA_{0.66}MA_{0.34}Pb_{0.50}Sn_{0.50}I_3$ PPD with an ITO top electrode and fitted Urbach tail. **c**, Temperature dependence of J_D calculated from the overlap integral between the Urbach tail or the entire EQE spectrum and the black-body photon flux for opaque (top Ag) and semi-transparent (top ITO) $FA_{0.66}MA_{0.34}Pb_{0.50}Sn_{0.50}I_3$ PPDs. Experimental J_D data points are shown as circles. **d**, EQE (grey dots, left scale) for a PTAA- $FA_{0.66}MA_{0.34}Pb_{0.5}Sn_{0.5}I_3$ PPD with ITO top and bottom electrodes (thus minimizing optical interference) and black-body photon flux at temperatures from 220 K to 340 K (yellow/brown, right scale). An Urbach tail has been fitted to the experimental EQE spectrum (black dashed line). Colored shaded areas show extrapolated low-energy features extensions to the observable EQE spectra that correspond to multiple hypothetical sub-bandgap trap state distributions. **e**, Temperature dependent J_D calculated from the overlap integral between each EQE spectrum (for the different hypothetical trap state distributions) and the black-body photon flux. Experimental J_D data points are again shown as circles – notably the calculation for each hypothetical EQE spectra fails to reproduce the temperature dependence of the experimental J_D.

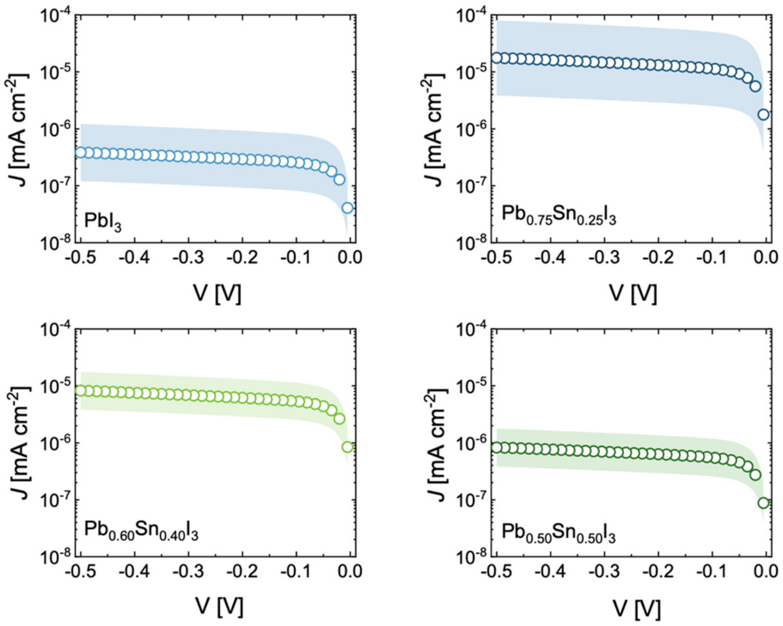

Supplementary Fig. 9. Simulated *J-V* curves for PTAA− $FA_{0.66}MA_{0.34}Pb_{(1-x)}Sn_xI_3$ PPDs with $x = 0$, 0.25, 0.40, 0.50, where thermally-activated charge generation at the EBL-perovskite interface is the dominant contribution to the current density due to the lowest energetic cost. Empty circles correspond to J_D resulting from the average energetic position of VBMs and CBMs, colored areas represent the uncertainty in the energy levels, as indicated in Supplementary Fig. 3.

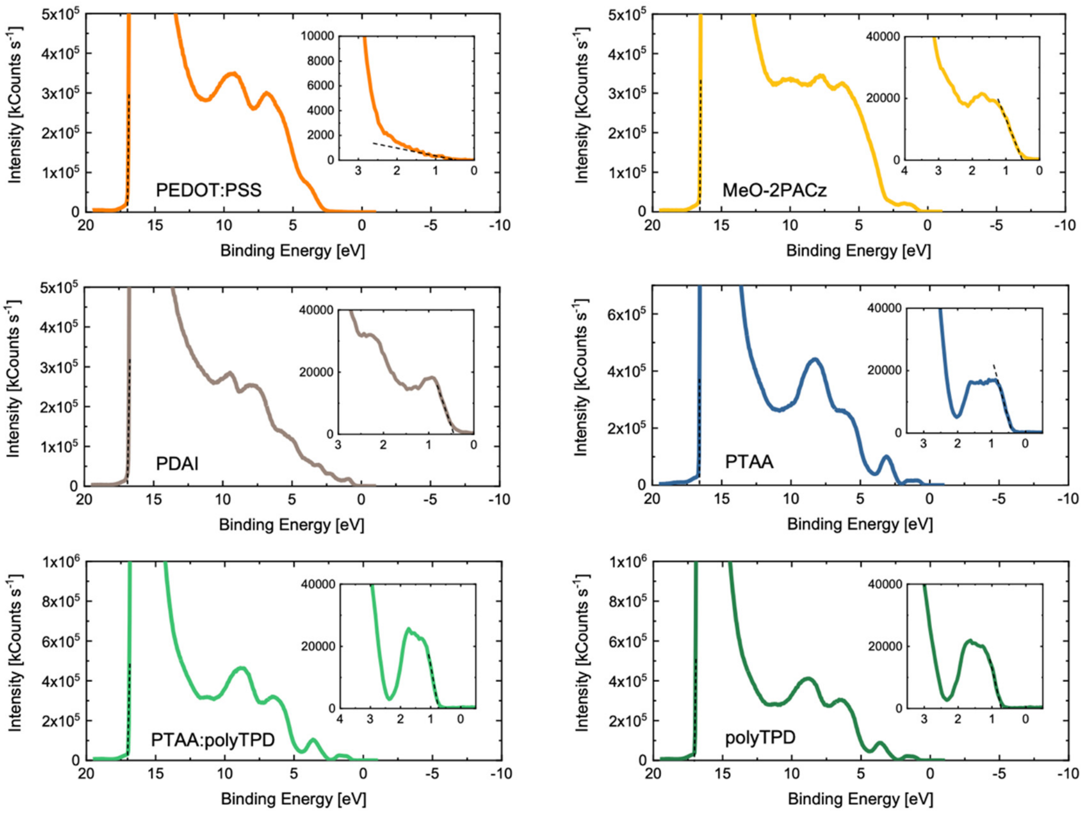

Supplementary Fig. 10. UPS measurements of EBLs. Spectra for PEDOT:PSS, MeO-2PACz, PDAI, PTAA, PTAA:poly-TPD, poly-TPD . The HOMO energy is determined as described for the VBM energy in the caption of Supplementary Fig. 3.

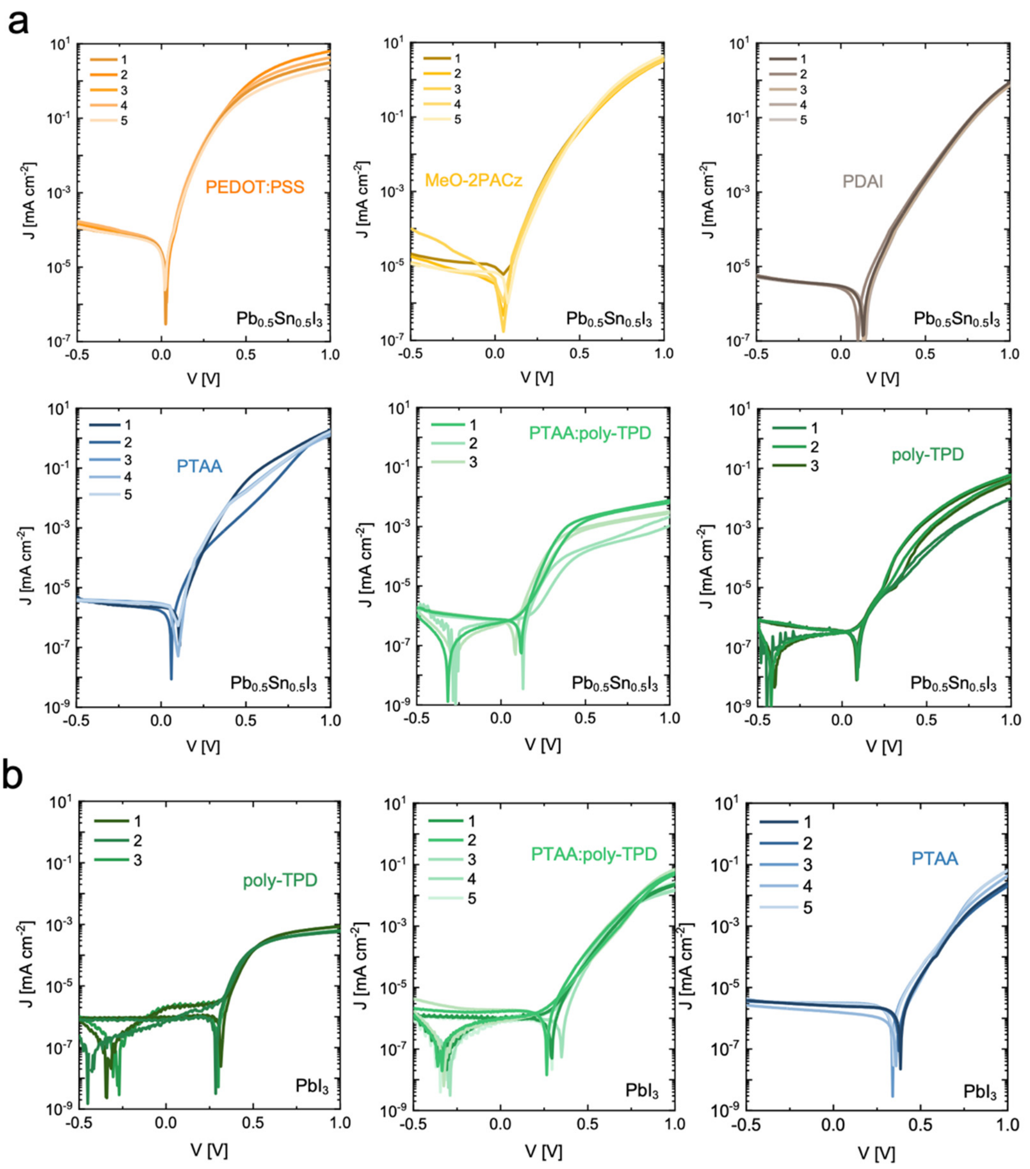

Supplementary Fig. 11. Reproducibility of *J-V* characteristics of PPDs. **a**, $FA_{0.66}MA_{0.34}Pb_{0.5}Sn_{0.5}I_3$. **b**, $FA_{0.66}MA_{0.34}PbI_3$. In each case the perovskite is combined with different EBL materials (PTAA, PDAI, PEDOT:PSS, MeO-2PACz, PTAA:poly-TPD and poly-TPD).

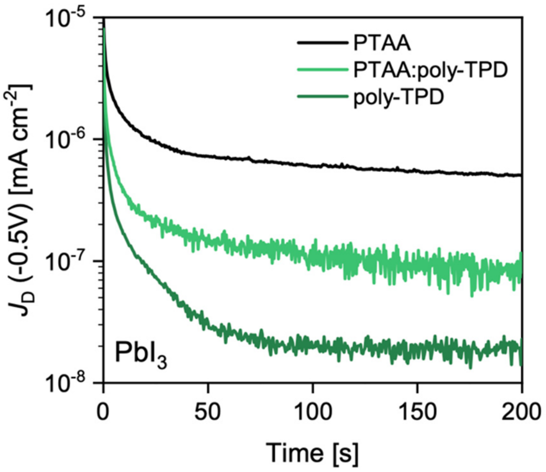

Supplementary Fig. 12. Time dependence of the current density for different EBL-$FA_{0.66}MA_{0.34}PbI_3$ perovskite PPDs. Traces were recorded by applying a constant bias of –0.5 V at room temperature.

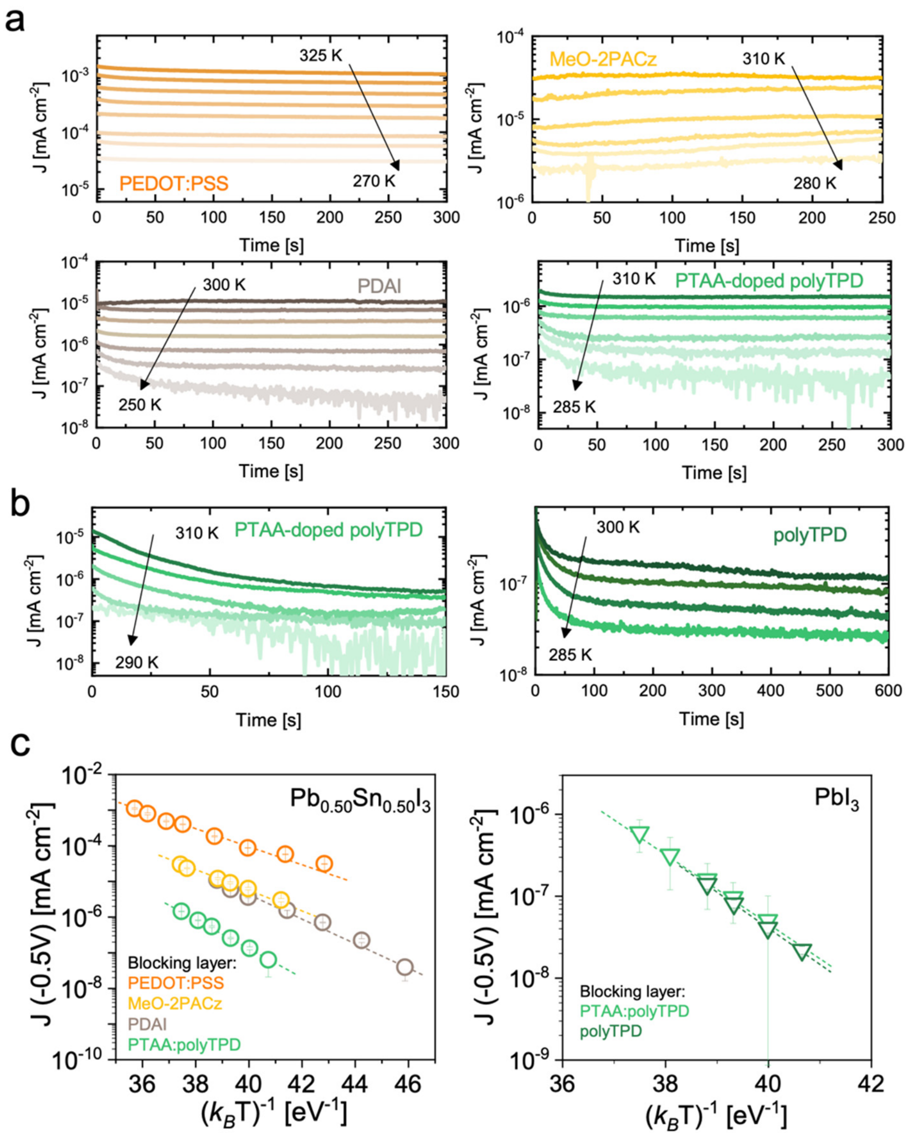

Supplementary Fig. 13. Time dependence of J_D as function of temperature. a, For EBL-$FA_{0.66}MA_{0.34}Pb_{0.5}Sn_{0.5}I_3$ PPD. **b**, EBL-$FA_{0.66}MA_{0.34}PbI_3$ PPD. Dark current density was recorded at $V = -0.5$ V until a constant value was reached. **c**, Temperature dependence of J_D at $V = -0.5$ V represented against the reciprocal temperature in an Arrhenius plot for different EBL-perovskite systems. Open symbols are experimental data extrapolated as mean value from the last 60 s of measurements shown in (**a**) and (**b**); whiskers represent standard deviation; dashed lines are linear fits.

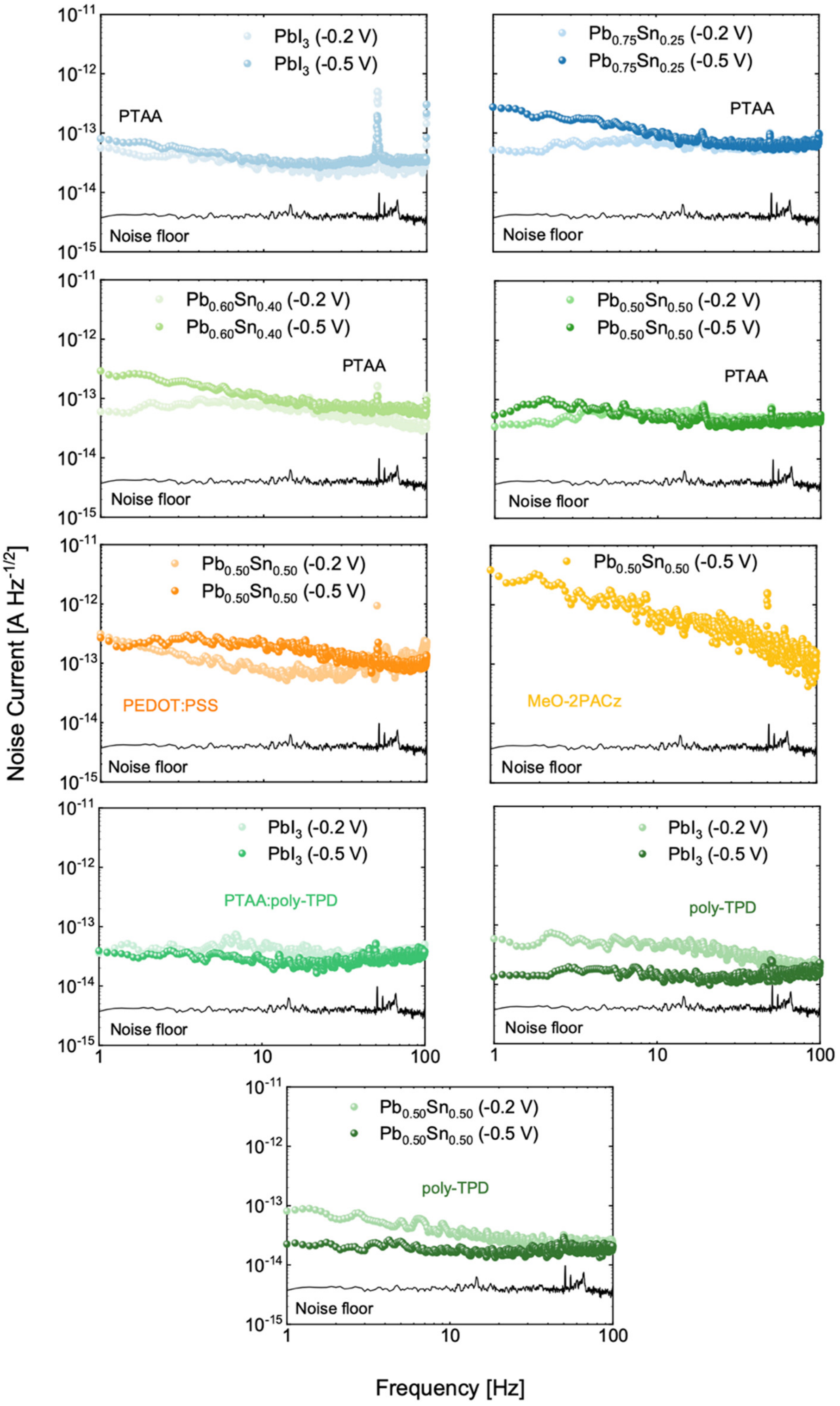

Supplementary Fig. 14. Noise current as function of frequency. Measured noise current vs. frequency under reverse bias (–0.5 and –0.2 V) for different EBL-perovskite systems, as indicated in the legend. Each spectrum is an average of 10 measurements. Harmonics with large amplitude in the spectrum are due to powerline interference. Black line represents the noise floor of the setup (~ 6 × 10^{-15} A $Hz^{-1/2}$).

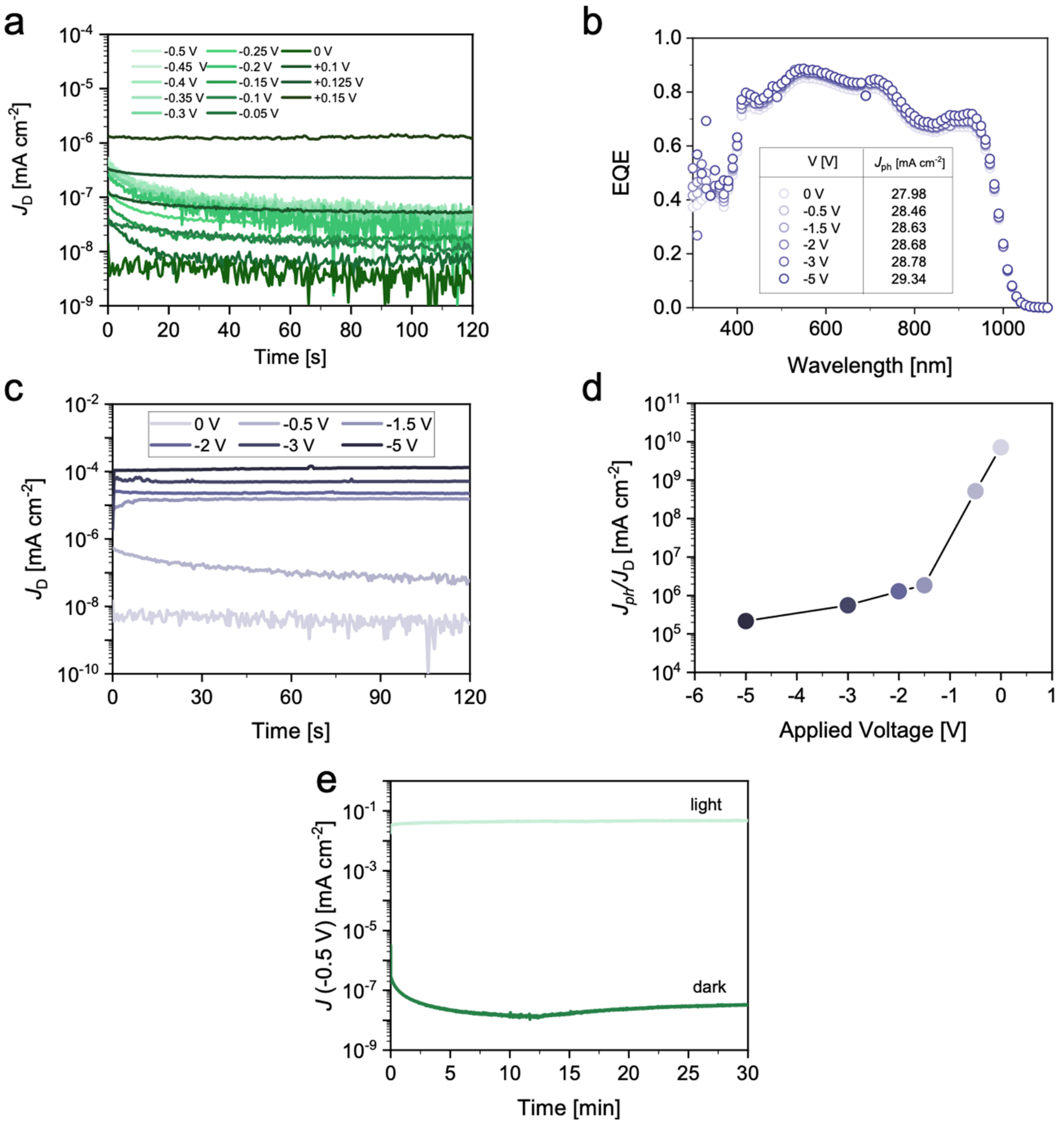

Supplementary Fig. 15. Dark and photocurrent density analysis of $FA_{0.66}MA_{0.34}Pb_{0.5}Sn_{0.5}I_3$ perovskite with a PTAA:poly-TPD EBL. a, Time dependence of the current density recorded by applying a constant bias voltage of –0.5 V to +0.15 V. **b**, External quantum efficiency (EQE) *vs.* wavelength measured at different reverse voltage bias. The corresponding J_{ph} (calculated from integrating the EQE spectrum with the solar AM1.5G spectrum) increases slightly with voltage, from 28.0 mA cm^{-2} at $V = 0$ V to 29.3 mA cm^{-2} at $V = -5$ V. **c**, Time dependence of the dark current density recorded by applying a reverse constant bias in the interval –5 V to –0.5 V. **d**, J_{ph}/J_D ratio as a function of applied voltage (using J_{ph} data from panel b and J_D data from panel c). **e**, Temporal stability of J_D and J_{ph}, measured for 30 min. in dark and light (60 µW cm^{-2}). A constant bias of –0.5 V was applied. The measurement was performed on the sample 12 months after its fabrication, showing it has good shelf life.

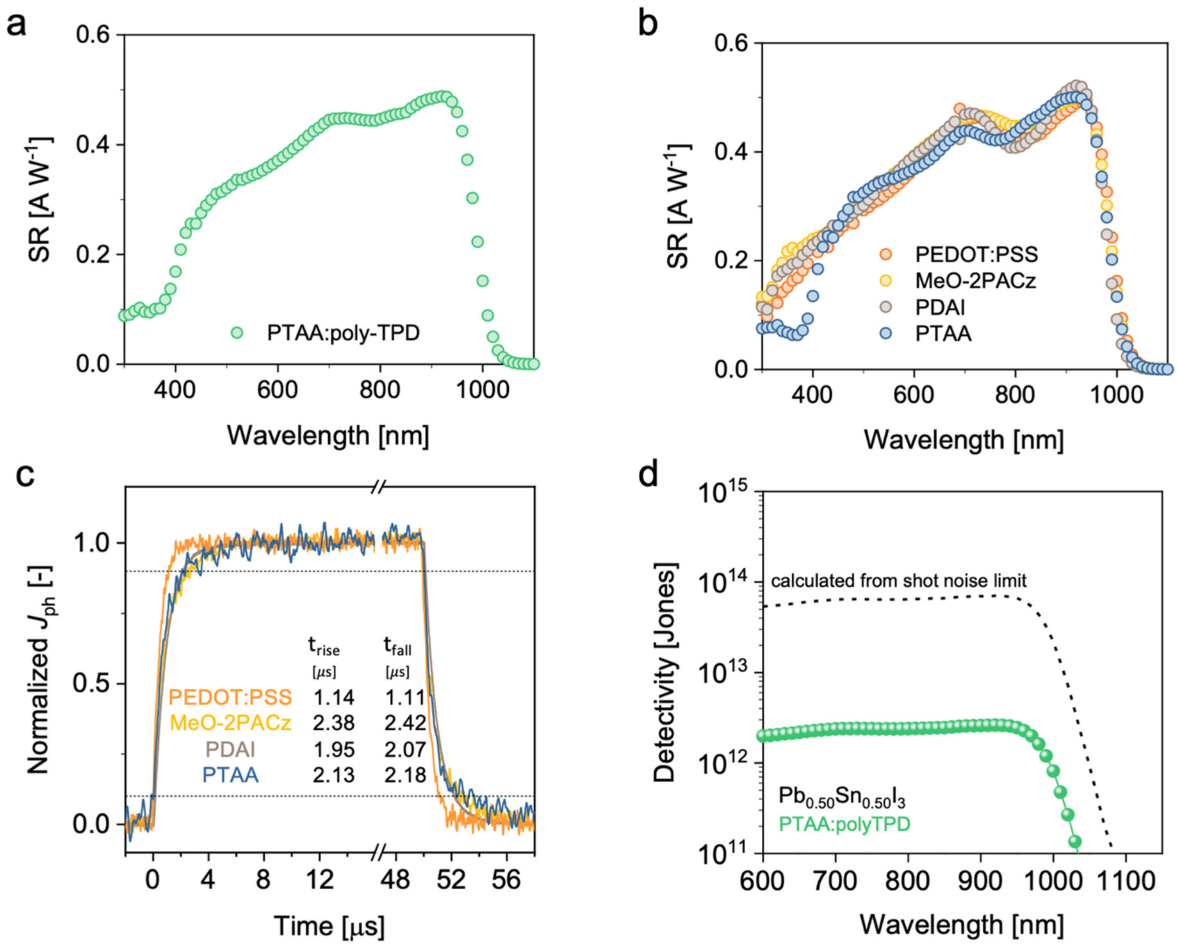

Supplementary Figure 16. Sensitivity of $FA_{0.66}MA_{0.34}Pb_{0.5}Sn_{0.5}I_3$ perovskite photodiode. a, Spectral responsivity (SR) *vs.* wavelength at –0.5 V for the PPD with PTAA:poly-TPD as EBL. **b**, SR *vs.* wavelength at –0.5 V for PPDs with other EBLs. **c**, Normalized transient photocurrent response of the device with different EBLs (540 nm light pulses of 50 μs duration). **d**, Detectivity calculated from dark current density using $D^* = \mathrm{SR} / (2qJ_\mathrm{D})^{1/2}$ and from measured noise current using $D^* = \mathrm{SR}\ \mathrm{A}^{1/2}/i_\mathrm{n}$ for photodiode with PTAA:poly-TPD EBL.

Supplementary Tables

Supplementary Table 1. Drift-diffusion simulation parameters used for Figure 1.

Material parameters	perovskite	PTAA	C_{60}
Thickness [nm]	600	5	20
Bandgap [eV]	1.23	3	1.6
ε_r	24.1	2.67	4.25
e^- thermal velocity [cm s^{-1}]	1×10^7	1×10^7	1×10^7
h^+ thermal velocity [cm s^{-1}]	1×10^7	1×10^7	1×10^7
μ_{e-} [cm^2 V^{-1} s^{-1}]	2.5	6×10^{-3}	1.6
μ_{h+} [cm^2 V^{-1} s^{-1}]	2.5	6×10^{-3}	1.6
CB effective DOS [cm^{-3}]	8.1×10^{18}	2.5×10^{19}	
VB effective DOS [cm^{-3}]	8.1×10^{18}	2.5×10^{19}	

Supplementary Table 2. Details of drift-diffusion simulation presented in Figure 2.

Contact material	WF [eV]	Thermionic emission / SRV [cm s^{-1}]	Effective mass of e^- (and h^+)
PTAA	5.2	1×10^7	0.2
C_{60}	4.2	1×10^7	0.2

Supplementary Table 3. Comparison between reported PPDs based on spectral sensitivity, dark current density (J_D), maximum spectral responsivity (R_{max}) and specific detectivity (D^*).

Reference	Spectral sensitivity [nm]	J_D [mA cm^{-2}]	R_{max} [A W^{-1}]	D^* [Jones][1]	Rise time [μs][2]	Fall time [μs][2]
[1]	up to ~800 nm	1×10^{-6} (−0.5 V)	0.34 (500 nm)	4×10^{12} (375-800 nm)*	~hundreds of ns[3]	~hundreds of ns[3]
[2]	up to ~800 nm	4×10^{-5} (−0.5 V)	0.4 (600 nm)	1×10^{12} (400-780 nm) †	1.2	3.2
[3]	up to ~800 nm	5×10^{-7} (−0.5 V)	–	2.9×10^{12} (700 nm) †	1.7	1
[4]	up to ~800 nm	2×10^{-7} (−0.2 V)	0.5 (750 nm)	4.5×10^{12} (450-750 nm)*	(0.058)	(0.168)
[5]	up to ~800 nm	1.75×10^{-6} (−1 V)	0.4 (640 nm)	2.7×10^{13} (800 nm)*	1.1	0.8
[6]	up to ~800 nm	5×10^{-6} (−0.5 V)	–	7.4×10^{12} (680 nm) †	–	(0.12)
[7]	up to ~950 nm	3.4×10^{-5} (−0.1 V)	0.37 (870 nm)	2.3×10^{11} (870 nm) †	35	20 (0.145)
[8]	up to ~1050 nm	1×10^{-3} (−0.4 V)	0.4 (800 nm)	3.25×10^{12} (800 nm) †	–	7.4
[9]	up to ~1000 nm	2×10^{-4} (−0.5 V)	0.45 (900 nm)	1×10^{12} (900 nm) †	6.9	9.1
This work	up to ~1050 nm	5×10^{-8} (−0.5 V)	0.5 (940 nm)	2.5×10^{12} †; 7×10^{13} (940 nm)*	0.79 (0.19)	0.88 (0.74)

[1] Specific detectivity derived from noise spectral density using $D^* = \mathrm{SR}\, A^{1/2}\, i_n^{-1}$ is marked with †. Specific detectivity values calculated using $D^* = \mathrm{SR}\,(2qJ_D)^{-1/2}$ (assuming dominant shot noise) are reported with *.

[2] The reported times are measured using a standard square wave and/or a short single peak pulse of light (represented in brackets).

[3] The reported times have been estimated based on mobilities of the materials used in the device and the thickness of each layer.

Supplementary Notes

Supplementary Note 1: Drift-diffusion simulations

The drift-diffusion simulations were performed using the SCAPS software version 3.3.07, developed by the group of Prof. Burgelman at the University of Gent. This software solves the continuity equation for electrons and holes together with the Poisson equation. Drift-diffusion simulations were employed to calculate the *J-V* characteristics of the PPDs in the reverse bias region (–0.5 V to 0 V) for several device configurations. Parameters used in these simulations are listed in Supplementary Table 1. To simulate the injection-dominant regime, the ITO/ perovskite/ C_{60}/ Ag stack was used, while for the injection-blocked regime (i.e., with EBL), a PTAA layer was included between ITO and perovskite. The energy levels were as reported in the band diagram of Figure 1 (main text).

To simulate the thermally-activated charge generation at the interface between the blocking layers and the perovskite, we treated the blocking layers as contacts under the assumption of no transport loss beyond their interface, as described by Wang et al.[10]. The simulated stack consisted then of PTAA/ perovskite/ C_{60}, with contacts parameters as reported in Supplementary Table 2. Perovskite properties were the same as previously listed in Supplementary Table 1, while the energetic information is reported in Figure 1.

Supplementary Note 2: Calculation temperature activation energies from sensitive EQE measurements

One possible source of J_D is thermally driven charge carrier generation within the bulk of the perovskite. This can occur with the excitation of carriers across the bandgap (i.e., from perovskite VB to CB), or between trap states that lie within the bandgap. To determine whether either of these mechanisms makes a significant contribution to J_D in the PPDs measured here, we adapt an approach commonly applied to calculate the short-circuit current density (J_{sc}) in solar cells that was also employed in our recently published work on the origins of OPD dark current[11,12]. More specifically, this widely used approach[13–15] proposes that J_{sc} is proportional to the overlap integral between the incident photon flux density $\phi(E)$ of the AM1.5G spectral radiance (determined from the spectral irradiance $I(E)$ via $\phi(E) = I(E)/E$ and the external quantum efficiency spectrum $EQE_{PV}(E)$ of charges collected per incident photon:

$$J_{\mathrm{sc}} = q\int_{E_1}^{E_2} \mathrm{EQE_{PV}}(E)\phi(E)dE \tag{S1}$$

The thermally generated dark current in the radiative limit J_0^{rad} can then be calculated by replacing the AM1.5G solar spectrum with the spectral photon flux from thermal black-body spectrum $\phi_{\mathrm{BB}}(E)$ at the device temperature:

$$J_0^{\mathrm{rad}} = q\int_0^{\infty} \mathrm{EQE_{PV}}(E)\phi_{\mathrm{BB}}(E)dE \tag{S2}$$

The calculated dark current ($J_{\mathrm{D}}^{\mathrm{calc}}$) then follows from $J_{\mathrm{D}}^{\mathrm{calc}} = \frac{J_0^{\mathrm{rad}}}{\mathrm{EQE_{EL}}}$, where $\mathrm{EQE_{EL}}$ is the absolute quantum efficiency of electroluminescence. The aforementioned implies that an Arrhenius type plot of J_0^{rad} calculated at different device temperatures would give an $E_{\mathrm{a}}^{\mathrm{EQE}}$ similar to the E_{a} experimentally measured J_{D} (assuming that only J_0^{rad} is temperature dependent).

The photon energy density $\rho(E)$ (units of m^{-3}) inside a black-body in the interval from E to $E + dE$, [14,16] is given by

$$\rho(E)dE = \frac{8\pi}{h^3c^3}\frac{E^3}{\exp\left(\frac{E}{k_{\mathrm{B}}T}\right)-1}dE, \tag{S3}$$

where c is the speed of light in a vacuum and k_{B} is Boltzmann's constant. Dividing by the energy of each photon E and multiplying by a factor of c gives the spectral photon flux $\phi(E)$ (in units of $\mathrm{J}^{-1}\ \mathrm{m}^{-2}\ \mathrm{s}^{-1}$) emitted into a hemisphere from a planar unit surface, again in the interval from E to $E + dE$, specifically

$$\phi(E)dE = \frac{2\pi}{h^3c^2}\frac{E^2}{\exp\left(\frac{E}{k_{\mathrm{B}}T}\right)-1}dE. \tag{S4}$$

Multiplying by a factor of two to account for the photon flux from both sides of the planar device (and converting to units of $\mathrm{eV}^{-1}\ \mathrm{cm}^{-2}\ \mathrm{s}^{-1}$) gives the spectral photon flux over at a certain temperature. Since for a black-body emission and absorption are equivalent, the thermally generated dark current density can thus be determined from Equation S1. This approach assumes that EQE is temperature invariant; which may not be perfectly true.

Determining whether charge carrier excitation between sub-bandgap trap states contributes to observed J_D requires extrapolation of the EQE spectrum to low energy regions that would correspond to plausible trap state distributions. Experimentally, we can determine the EQE down to ~0.63 eV, leaving the spectral shape below this energy, or when the limits of experimental sensitivity are reached, undetermined. Furthermore, because the black-body photon flux spectrum at room temperature and below is concentrated at low energies (*e.g.*, maximum flux is at ~0.2 eV at 300 K), ignoring photon energies below 0.63 eV when determining EQE_{PV} will underestimate J_D^{calc}[17].

Supplementary references

1. Liu, C. et al. Ultrasensitive solution-processed perovskite hybrid photodetectors. *J. Mater. Chem. C* **3**, 6600–6606 (2015).

2. Sutherland, B. R. et al. Sensitive, Fast, and Stable Perovskite Photodetectors Exploiting Interface Engineering. *ACS Photonics* **2**, 1117–1123 (2015).

3. Lin, Q., Armin, A., Lyons, D. M., Burn, P. L. & Meredith, P. Low noise, IR-blind organohalide perovskite photodiodes for visible light detection and imaging. *Adv. Mater.* **27**, 2060–2064 (2015).

4. Zhu, H. L. et al. Room-Temperature Solution-Processed NiO_x:PbI_2 Nanocomposite Structures for Realizing High-Performance Perovskite Photodetectors. *ACS Nano* **10**, 6808–6815 (2016).

5. Wang, Y. et al. $CH_3NH_3PbI_3/C_{60}$ heterojunction photodetectors with low dark current and high detectivity. *Org. Electron.* **42**, 203–208 (2017).

6. Fang, Y. & Huang, J. Resolving weak light of sub-picowatt per square centimeter by hybrid perovskite photodetectors enabled by noise reduction. *Adv. Mater.* **27**, 2804–2810 (2015).

7. Li, C. et al. Ultrafast and broadband photodetectors based on a perovskite/organic bulk heterojunction for large-dynamic-range imaging. *Light Sci. Appl.* **9**, 31 (2020).

8. Xu, X. et al. High-Performance Near-IR Photodetector Using Low-Bandgap $MA_{0.5}FA_{0.5}Pb_{0.5}Sn_{0.5}I_3$ Perovskite. *Adv. Funct. Mater.* **27**, 1701053 (2017).

9. Wang, W. et al. Highly Sensitive Low-Bandgap Perovskite Photodetectors with Response from Ultraviolet to the Near-Infrared Region. *Adv. Funct. Mater.* **27,** 1703953 (2017).

10. Wang, J. et al. Reducing Surface Recombination Velocities at the Electrical Contacts Will Improve Perovskite Photovoltaics. *ACS Energy Lett.* **4**, 222–227 (2019).

11. Simone, G. et al. On the Origin of Dark Current in Organic Photodiodes. *Adv. Opt. Mater.* **8**, 1901568 (2020).

12. Simone, G., Dyson, M. J., Meskers, S. C. J., Janssen, R. A. J. & Gelinck, G. H. Organic Photodetectors and their Application in Large Area and Flexible Image Sensors: The Role of Dark Current. *Adv. Funct. Mater.* **30**, 1904205 (2020).

13. Liang, Y. et al. For the bright future-bulk heterojunction polymer solar cells with power conversion efficiency of 7.4%. *Adv. Mater.* **22**, 135–138 (2010).

14. Vandewal, K., Tvingstedt, K., Manca, J. V. & Inganäs, O. Charge-transfer states and upper limit of the open-circuit voltage in polymer: Fullerene oprganic solar cells. *IEEE J. Sel. Top. Quantum Electron.* **16**, 1676–1684 (2010).

15. Padilla, M., Michl, B., Thaidigsmann, B., Warta, W. & Schubert, M. C. Short-circuit current density mapping for solar cells. *Sol. Energy Mater. Sol. Cells* **120**, 282–288 (2014).

16. Olson, K. D. & Talghader, J. J. Absorption to reflection transition in selective solar coatings. *Opt. Express* **20**, 26744 (2012).

17. Peaker, A. R., Markevich, V. P. & Coutinho, J. Junction spectroscopy techniques and deep-level defects in semiconductors. *J. Appl. Phys.* **123**, 161559 (2018).
